# NCF1-dependent production of ROS protects against lupus by regulating plasmacytoid dendritic cell development and functions

**DOI:** 10.1172/jci.insight.164875

**Published:** 2023-04-10

**Authors:** Huqiao Luo, Vilma Urbonaviciute, Amir Ata Saei, Hezheng Lyu, Massimiliano Gaetani, Ákos Végvári, Yanpeng Li, Roman A. Zubarev, Rikard Holmdahl

**Affiliations:** 1 Division of Medical Inflammation Research and; 2 Division of Physiological Chemistry I, Department of Medical Biochemistry and Biophysics, Karolinska Institutet, Stockholm, Sweden.; 3 Department of Cell Biology, Harvard Medical School, Boston, Massachusetts, USA.; 4 SciLifeLab, Stockholm, Sweden.; 5 Chemical Proteomics Core Facility and; 6 Proteomics Biomedicum, Division of Physiological Chemistry I, Department of Medical Biochemistry and Biophysics, Karolinska Institutet, Stockholm, Sweden.

**Keywords:** Autoimmunity, Dendritic cells, Lupus

## Abstract

Low capacity to produce ROS because of mutations in neutrophil cytosolic factor 1 (NCF1/p47phox), a component of NADPH oxidase 2 (NOX2) complex, is strongly associated with systemic lupus erythematosus in both humans and mouse models. Here, we aimed to identify the key immune cell type(s) and cellular mechanisms driving lupus pathogenesis under the condition of NCF1-dependent ROS deficiency. Using cell-specific Cre-deleter, human *NCF1*-339 variant knockin, and transgenic mouse strains, we show that low ROS production in plasmacytoid dendritic cells (pDCs) exacerbated both pristane-induced lupus and a potentially new Y-linked autoimmune accelerating locus–related spontaneous model by promoting pDC accumulation in multiple organs during lupus development, accompanied by elevated IFN-α levels and expression of IFN-stimulated genes. Mechanistic studies revealed that ROS deficiency enhanced pDC generation through the AKT/mTOR pathway and CCR2-mediated migration to tissues, which together with hyperactivation of the redox-sensitive stimulator of interferon genes/IFN-α/JAK1/STAT1 cascade further augmented type I IFN responses. More importantly, by suppressing these pathways, restoration of NOX2-derived ROS specifically in pDCs protected against lupus. These discoveries explain the causative effect of dysfunctional NCF1 in lupus and demonstrate the protective role of pDC-derived ROS in disease development driven by NCF1-dependent ROS deficiency.

## Graphical abstract

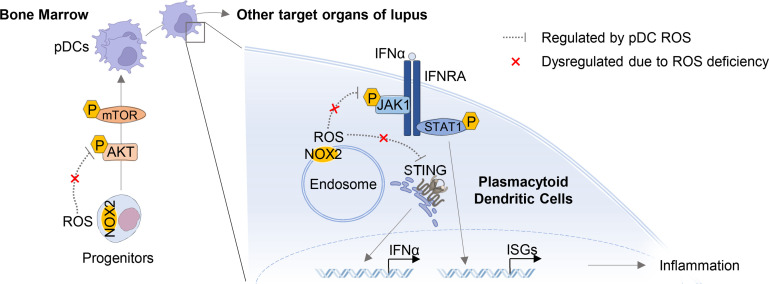

## Introduction

Systemic lupus erythematosus (SLE) is a chronic systemic autoimmune disease with a global incidence of around 0.1%, predominantly observed in women of child-bearing age (1). Patients with SLE present a broad range of clinical manifestations, including skin rash, arthritis, glomerulonephritis, neurological symptoms, hemolytic anemia, and vasculitis (2). The disease is characterized by loss of tolerance to ubiquitous self-antigens, high titers of autoantibodies directed against nuclear components such as dsDNA and nucleosomes, immune complex (IC) deposition, as well as involvement of multiple organs. Organ damage occurs as a consequence of the chronic, uncontrolled autoimmune responses, or secondarily because of vasculitis- and thrombosis-induced ischemia. The majority of patients with SLE display overactivation of the type I IFN system, a so-called type I IFN signature, indicated by increased serum level of IFN-α and expression of type I IFN-stimulated genes (ISGs), which correlates with SLE activity and severity (1). IFNs act as an immune adjuvant for diverse immune responses, including production of proinflammatory cytokines and autoantibodies by stimulating monocytes, T cells, and B cells (3). The exact cause of SLE has not been fully understood, yet it is believed that genetic predisposition together with exogenous factors such as viral infections, smoking, UV irradiation, and certain drugs lead to dysregulation of innate and adaptive immune systems, subsequently driving the clinical disease (4).

The regulatory role of ROS has been recently considered key to development of inflammatory and autoimmune diseases such as SLE (1, 5). Mutations of genes determining NADPH oxidase 2 (NOX2) complex, a membrane enzyme complex in endothelial and phagocytic cells with primary function of generating ROS in response to innate immune stimuli, lead to chronic granulomatous disease (CGD), which is accompanied by various autoimmune manifestations, including inflammatory bowel disease, lupus, antiphospholipid syndrome, and polyarthritis (5). Of importance, an SNP in the neutrophil cytosolic factor 1 (*NCF1*) gene, which encodes the NCF1 protein (also known as p47phox), a key subunit of NOX2, has been identified (denoted *NCF1*-339) (6). The dysfunctional *NCF1*-339 allele, leading to impaired function of NOX2 complex, represents the strongest genetic association for SLE, regarding both odds ratio and allelic frequency (1, 7, 8).

Previous studies have confirmed a role of NCF1-dependent ROS in mouse models of SLE (9–12). Mice with low ROS production due to the *Ncf1^m1j^* mutation spontaneously develop elevated levels of lupus-associated autoantibodies, significant deposits of IgG and complement C3 in glomeruli, as well as high expression of ISGs and inflammation-related genes (5, 13). Similar lupus-like manifestations are observed in naive mice carrying the human SNP *NCF1*-339, which can be exacerbated by pristane injection (9). Several mechanisms have been proposed for the association between NCF1-derived ROS deficiency and lupus development. Impaired formation of neutrophil extracellular traps (NETs) and insufficient efferocytosis by macrophages/monocytes because of reduced endosomal acidification are suggested to contribute to aggravation of pristane-induced lupus (PIL) in ROS-deficient mice (9, 11, 14). In addition, acidic pH in endosomes caused by defective localization of NCF1 activates TLR signaling in plasmacytoid dendritic cells (pDCs), the principal producer of type I IFNs, leading to increased IFN secretion and thereafter more severe disease in the imiquimod-induced lupus model (12, 15–17). Nevertheless, the key immune cell type(s) mediating NCF1-dependent lupus exacerbation and underlying mechanisms require elucidation.

In this study, using cell-specific Cre-deleter, knockin, and transgenic mouse strains, we systematically investigated which immune cell type(s) are responsible for ROS-dependent lupus exacerbation. We identified pDCs as the pathogenic cells in PIL and a potentially new spontaneous lupus model. ROS deficiency due to the *Ncf1* mutation or the *NCF1*-339 SNP enhanced pDC generation through the AKT/mTOR pathway and CCR2-dependent pDC accumulation in multiple organs during disease development. Increased numbers of pDCs, together with their hyperactivation via stimulator of interferon genes (STING)/IFN-α/JAK1/STAT1 signaling, resulted in higher IFN-α levels and ISG expression, explaining the impact of NCF1-derived ROS on lupus severity.

## Results

### Deficiency in NOX2-derived ROS leads to PIL exacerbation in Ncf1-mutant mice.

**Figure 1:**
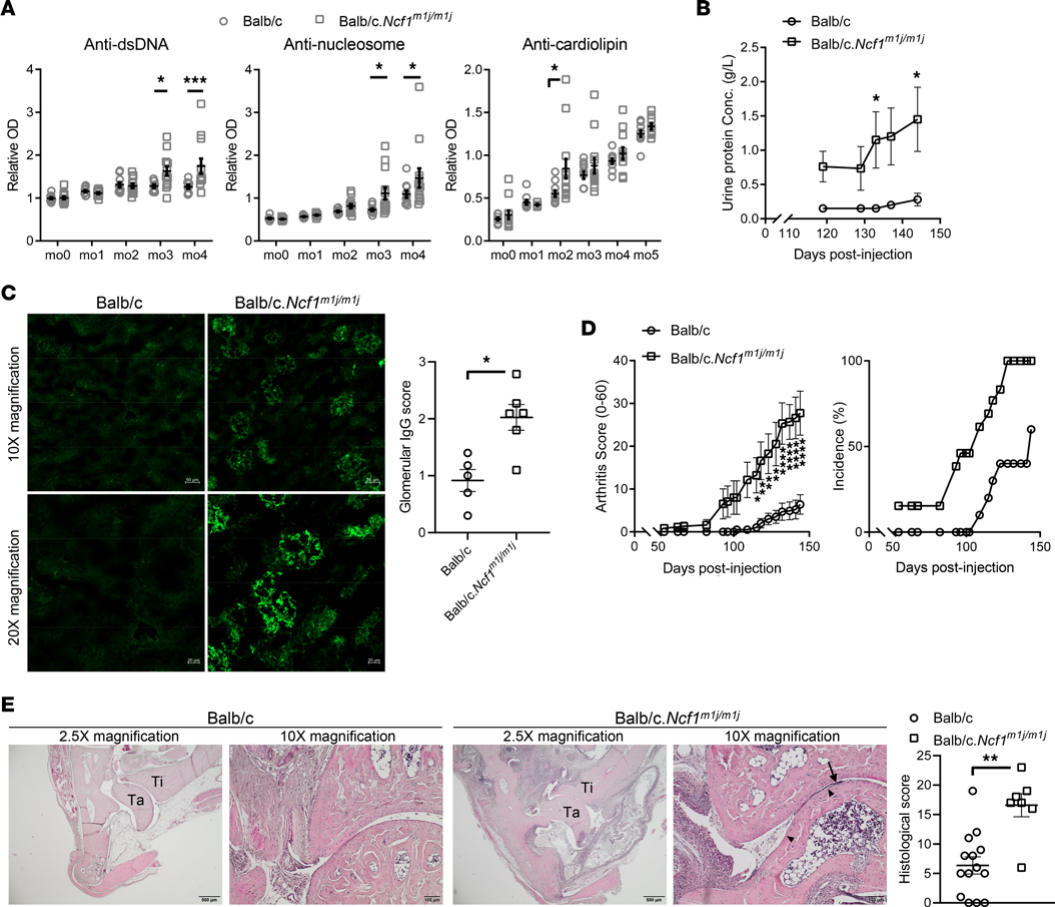
Deficiency in NOX2-derived ROS leads to PIL exacerbation in *Ncf1*-mutant mice. (**A**) Serum levels of autoantibodies against dsDNA, nucleosomes, and cardiolipin; (**B**) urinary protein concentrations; and (**D**) clinical scores and incidence of PIL in Balb/c.*Ncf1^m1j/m1j^* (*n* = 15) and Balb/c WT littermates (*n* = 10) at different time points after pristane injection. (**C**) The fluorescence intensity of IgG within glomeruli from Balb/c.*Ncf1^m1j/m1j^* (*n* = 6) and Balb/c WT littermates (*n* = 5) at 5 MPI. Representative images of IgG deposits are taken at original magnification 10× (scale bar = 50 μm) and 20× (scale bar = 20 μm). (**E**) Histopathological scoring of joints from Balb/c.*Ncf1^m1j/m1j^* (*n* = 7) and Balb/c WT littermates (*n* = 14) at 5 MPI. Representative sections of joints are taken at original magnification 2.5× (scale bar = 500 μm) and 10× (scale bar = 100 μm) (Ti, tibia; Ta, talus). Pannus and bone erosion are indicated by the arrow and triangles, respectively. Statistical significance is determined by 2-way ANOVA with Holm-Šídák multiple-comparison test (**A**, **B**, and **D**) and 2-tailed Mann-Whitney *U* test (**C** and **E**). Significance is presented as asterisks (*P < 0.05, **P < 0.01, ***P < 0.001, ****P < 0.0001).

*Ncf1^m1j/m1j^* mice carry a loss-of-function mutation in the *Ncf1* gene, which encodes an essential subunit of the NOX2 complex, leading to complete inhibition of ROS production (13). It has been reported that *Ncf1^m1j/m1j^* on Balb/c background develop exacerbated PIL compared with Balb/c WT, as represented by more severe IC glomerulonephritis and higher levels of lupus-associated autoantibodies (11, 18). In agreement with the previous studies, here we show that Balb/c.*Ncf1^m1j/m1j^* produced higher levels of autoantibodies against dsDNA and nucleosomes (Figure 1A). An increase in anti-cardiolipin antibody production was also observed at 2 months post pristane injection (MPI) (Figure 1A). Moreover, Balb/c.*Ncf1^m1j/m1j^* mice displayed more severe and accelerated kidney disease, reflected by higher and earlier appearing urinary protein excretion (Figure 1B) and more frequent glomerular IgG deposits in the kidneys (Figure 1C). PIL on Balb/c background presents lupus arthritis similar to the manifestations in patients with SLE (18). We found that the pristane-treated Balb/c.*Ncf1^m1j/m1j^* mice developed earlier and more severe lupus arthritis with higher incidence compared with WT, as evidenced by clinical and histopathological assessments (Figure 1, D and E). Taken together, deficiency of NOX2-derived ROS causes more severe lupus-like disease.

### ROS deficiency in Mrp8^+^ cells, mainly neutrophils, does not affect PIL development.

**Figure 2:**
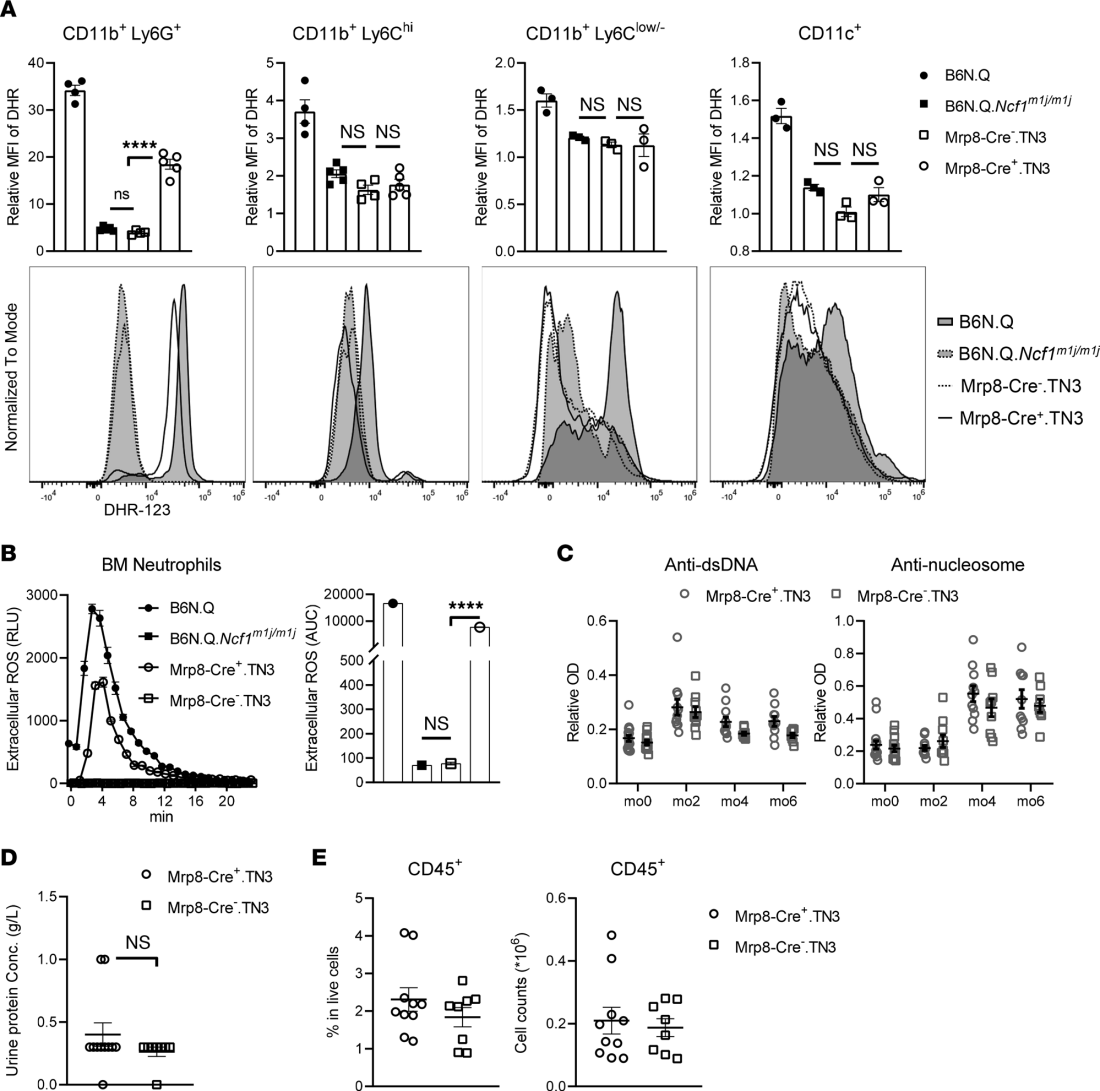
ROS deficiency in Mrp8^+^ cells, mainly neutrophils, does not affect PIL development. (**A**) Oxidative burst in neutrophils and monocytes within peripheral blood from naive B6N.Q (*n* = 4), B6N.Q.*Ncf1^m1j/m1j^* (*n* = 5), Mrp8-Cre^–^.TN3 (*n* = 5), and Mrp8-Cre^+^.TN3 mice (*n* = 4). Oxidative burst in macrophages and DCs within spleen from naive B6N.Q, B6N.Q.*Ncf1^m1j/m1j^*, Mrp8-Cre^–^.TN3, and Mrp8-Cre^+^.TN3 (of each *n* = 3). (**B**) Extracellular ROS in BM neutrophils from naive B6N.Q, B6N.Q.*Ncf1^m1j/m1j^*, Mrp8-Cre^–^.TN3, and Mrp8-Cre^+^.TN3 mice (of each *n* = 3). AUCs are calculated and plotted. (**C**) Serum levels of autoantibodies against dsDNA and nucleosomes in Mrp8-Cre^+^.TN3 (*n* = 10) and Mrp8-Cre^–^.TN3 mice (*n* = 8) at different time points after pristane injection. (**D**) Urinary protein concentrations and (**E**) frequency and numbers of CD45^+^ cells within kidneys from Mrp8-Cre^+^.TN3 (*n* = 10) and Mrp8-Cre^–^.TN3 mice (*n* = 8) at 6 MPI. Statistical analysis is done by 1-way ANOVA with Tukey’s multiple-comparison test (**A** and **B**), 2-way ANOVA with Holm-Šídák multiple-comparison test (**C**), and 2-tailed Mann-Whitney *U* test (**D** and **E**). ****P < 0.0001. DHR, dihydrorhodamine 123.

Neutrophils express a high level of NOX2 complex, leading to strong respiratory burst upon stimulation, and they have been suggested to be important in lupus development (11). To investigate the role of NOX2-derived ROS from neutrophils in lupus, we used a mouse strain expressing NCF1 under the control of the human S100A8 promoter, Mrp8-Cre.TN3. Oxidative burst was detected only in neutrophils but not in macrophages/monocytes or DCs from B6N.Q.Mrp8-Cre^+^.TN3 (Mrp8-Cre^+^.TN3) mice (Figure 2A). The capacity of extracellular ROS production in neutrophils was partially restored (Figure 2B). Considering that S100A8 can be upregulated during infection and cytokine stimulation in cells other than neutrophils, we measured oxidative burst in various peripheral blood cell types from Mrp8-Cre.TN3 strains before and after pristane injection (19). Only neutrophils displayed ROS burst, confirming the specificity of this strain (Supplemental Figure 1; supplemental material available online with this article; https://doi.org/10.1172/jci.insight.164875DS1).

We injected Mrp8-Cre.TN3 mice with pristane and followed the development of lupus. Mrp8-Cre^+^.TN3 and Mrp8-Cre^–^.TN3 mice developed comparable levels of autoantibodies against dsDNA and nucleosomes during the disease (Figure 2C). Similar levels of urinary protein and renal infiltration of CD45^+^ immune cells were observed at 6 MPI between Mrp8-Cre^+^.TN3 mice and their Cre^–^ littermates (Figure 2, D and E), indicating comparable kidney disease severity. The results suggest that NOX2-derived ROS from neutrophils are dispensable in PIL.

### NOX2-derived ROS in CD68^+^ cells, representing monocytes/macrophages and pDCs, protect against PIL.

**Figure 3:**
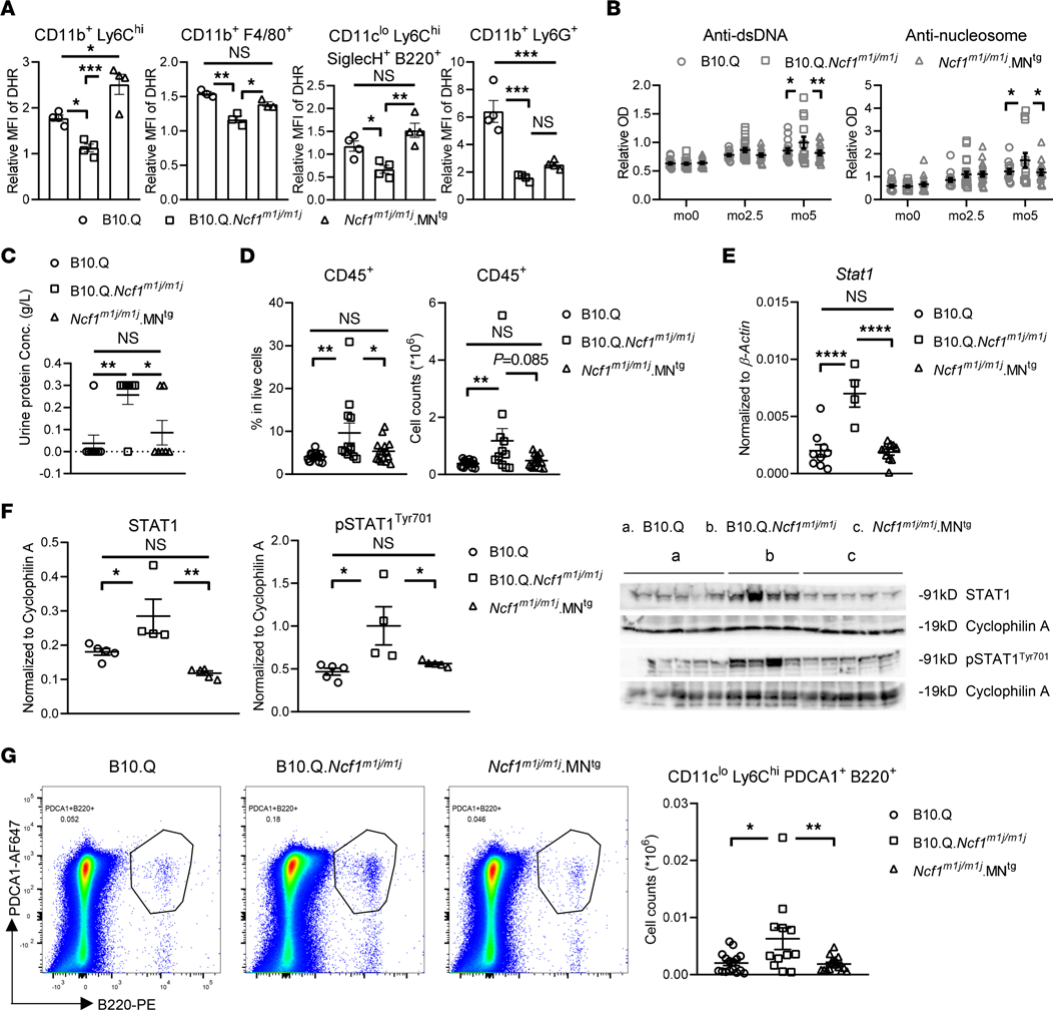
NOX2-derived ROS in CD68^+^ cells, representing monocytes/macrophages and pDCs, protect against PIL. (**A**) Oxidative burst in monocytes, pDCs, and neutrophils within peripheral blood from naive B10.Q, B10.Q. *Ncf1^m1j/m1j^*, and *Ncf1^m1j/m1j^*.MN^Tg^ (*n* = 4) and peritoneal macrophages from naive B10.Q, B10.Q.*Ncf1^m1j/m1j^*, and *Ncf1^m1j/m1j^*.MN^Tg^ (*n* = 3). (**B**) Serum levels of autoantibodies against dsDNA and nucleosomes in B10.Q (*n* = 18), B10.Q.*Ncf1^m1j/m1j^* (*n* = 22), and *Ncf1^m1j/m1j^*.MN^Tg^ (*n* = 18) at different time points after pristane injection. (**C**) Urinary protein concentrations in B10.Q (*n* = 8), B10.Q.*Ncf1^m1j/m1j^* (*n* = 8), and *Ncf1^m1j/m1j^*.MN^Tg^ (*n* = 7) at 5 MPI. (**D**) Frequency and numbers of CD45^+^ cells within kidneys from B10.Q (*n* = 16), B10.Q.*Ncf1^m1j/m1j^* (*n* = 12), and *Ncf1^m1j/m1j^*.MN^Tg^ (*n* = 15) at 7 MPI. (**E**) mRNA level of STAT1 within kidneys from B10.Q (*n* = 9), B10.Q.*Ncf1^m1j/m1j^* (*n* = 4), and *Ncf1^m1j/m1j^*.MN^Tg^ (*n* = 10) at 7 MPI. (**F**) Protein level of STAT1 and pSTAT1^Tyr701^ within kidneys from B10.Q (*n* = 5), B10.Q.*Ncf1^m1j/m1j^* (*n* = 4), and *Ncf1^m1j/m1j^*.MN^Tg^ (*n* = 5) at 7 MPI. Quantification of bands normalized to cyclophilin A is presented. (**G**) Numbers of pDCs within kidneys from B10.Q (*n* = 16), B10.Q.*Ncf1^m1j/m1j^* (*n* = 12), and *Ncf1^m1j/m1j^*.MN^Tg^ (*n* = 15) at 7 MPI. Representative flow cytometry plots are presented. Statistical significance is determined by 1-way ANOVA with Tukey’s multiple-comparison test (**A** and **C**–**G**) and 2-way ANOVA with Tukey’s multiple-comparison test (**B**). *P < 0.05, **P < 0.01, ***P < 0.001, ****P < 0.0001.

Having excluded the role of neutrophil-specific NOX2-derived ROS in PIL, we investigated the effect of ROS produced by myeloid and dendritic cells, which express levels of NOX2 second only to neutrophils (20). We used the B10.Q.*Ncf1^m1j/m1j^*.MN^Tg^ (*Ncf1^m1j/m1j^*.MN^Tg^) mouse strain, which expresses functional NCF1 protein under the control of human CD68 promoter. In humans and mice, CD68 is highly expressed on monocytes/macrophages and pDCs (20) (Supplemental Figure 2A). Oxidative burst assays verified ROS restoration in these cells but not neutrophils from *Ncf1^m1j/m1j^*.MN^Tg^ mice (Figure 3A).

With restored NOX2-derived ROS in monocytes/macrophages and pDCs, *Ncf1^m1j/m1j^*.MN^Tg^ mice developed milder lupus-like disease after pristane injection than *Ncf1^m1j/m1j^* mice. Comparable levels of autoantibodies against dsDNA and nucleosomes were found in the sera from *Ncf1^m1j/m1j^*.MN^Tg^ and WT mice during PIL (Figure 3B). In addition, similar levels of urinary protein and numbers of CD45^+^ cells in kidneys were observed between *Ncf1^m1j/m1j^*.MN^Tg^ and WT mice at 7 MPI (Figure 3, C and D).

Since type I IFNs are central and reflective of lupus severity and activity, we wondered if NOX2-derived ROS ameliorate kidney damage in *Ncf1^m1j/m1j^*.MN^Tg^ mice by dampening IFN responses. We measured expression and phosphorylation of STAT1 (p-STAT1), one of the key transcriptional factors activating ISG expression. Higher STAT1 and p-STAT1 were found in the kidneys of *Ncf1^m1j/m1j^* mice compared with WT mice (Figure 3, E and F). Of importance, we observed comparable levels of STAT1 and p-STAT1 in the kidneys of *Ncf1^m1j/m1j^*.MN^Tg^ and WT mice (Figure 3, E and F). Consistent with IFN signaling, we found more pDCs, the main producer of type I IFNs, infiltrating kidneys of *Ncf1^m1j/m1j^* mice compared with WT mice and a similar number between *Ncf1^m1j/m1j^*.MN^Tg^ and WT mice (Figure 3G). In addition to kidneys, we observed the same pattern in the spleen at 7 MPI (Supplemental Figure 2B). These results suggest that ROS produced by monocytes/macrophages and pDCs protect against PIL by controlling pDC accumulation at inflammatory sites and in lymphoid organs, thereby limiting IFN responses during disease development.

### Restoration of ROS in CD11c^+^ cells, predominantly DCs, alleviates PIL.

**Figure 4:**
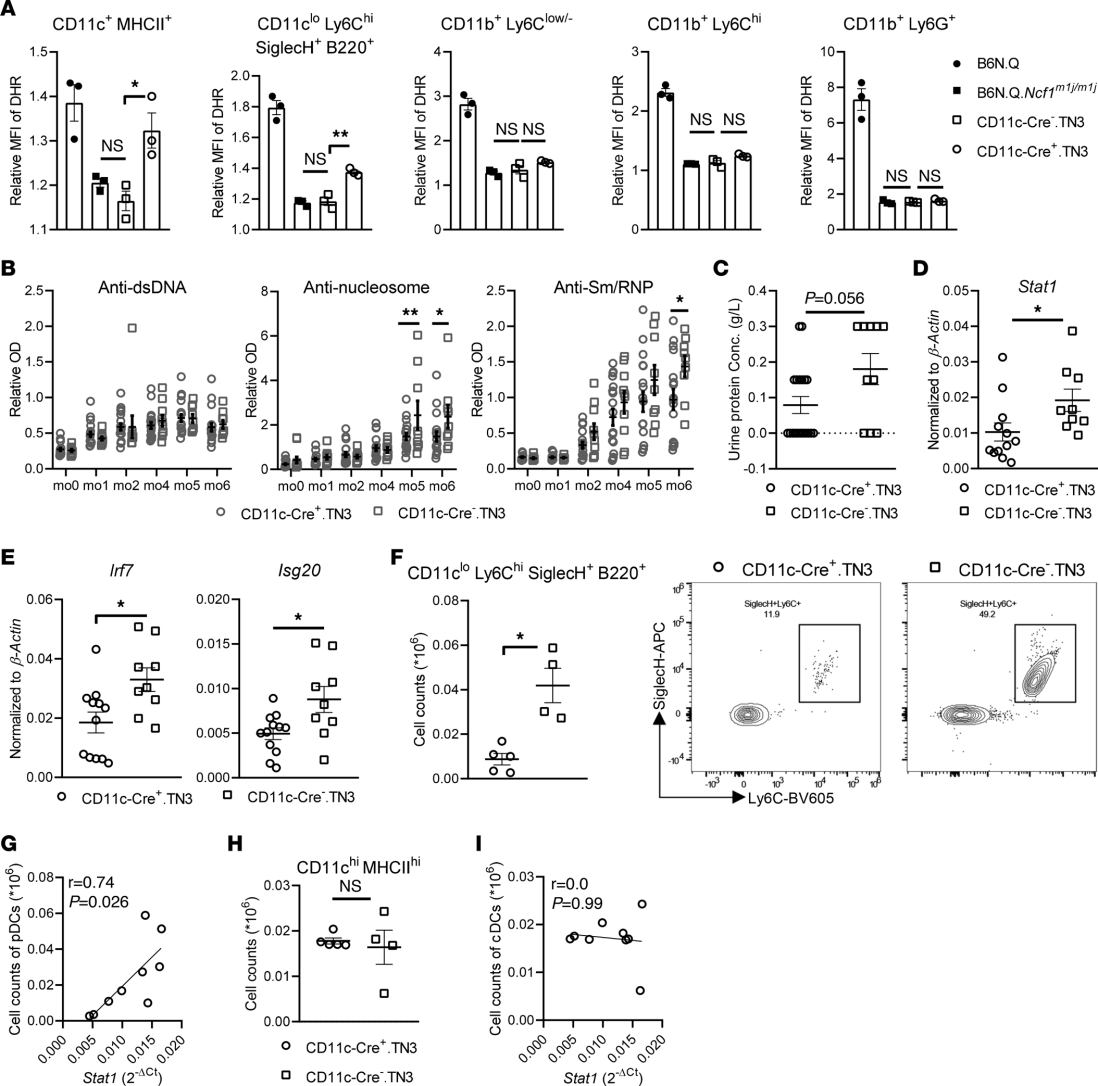
Restoration of ROS in CD11c^+^ cells, predominantly DCs, alleviates PIL. (**A**) Oxidative burst in cDCs, pDCs, macrophages, monocytes, and neutrophils within spleen from naive B6N.Q, B6N.Q.*Ncf1^m1j/m1j^*, CD11c-Cre^–^.TN3, and CD11c-Cre^+^.TN3 mice (of each *n* = 3). (**B**) Serum levels of autoantibodies against dsDNA, nucleosomes, and Sm/RNP in CD11c-Cre^+^.TN3 (*n* = 21) and CD11c-Cre^–^.TN3 mice (*n* = 11) at time points after pristane injection. (**C**) Urinary protein concentrations in CD11c-Cre^+^.TN3 (*n* = 19) and CD11c-Cre^–^.TN3 mice (*n* = 10) at 6 MPI. (**D**) mRNA level of STAT1 and (**E**) ISGs within kidneys from CD11c-Cre^+^.TN3 (*n* = 12) and CD11c-Cre^–^.TN3 mice (*n* = 9) at 7 MPI. (**F**) Numbers of pDCs within kidneys from CD11c-Cre^+^.TN3 (*n* = 5) and CD11c-Cre^–^.TN3 (*n* = 4) male mice at 7 MPI. Representative flow cytometry plots are presented. (**G**) Correlation between numbers of pDCs and mRNA level of STAT1 within kidneys from CD11c-Cre^+^.TN3 and CD11c-Cre^–^.TN3 males. (**H**) Numbers of cDCs within kidneys from CD11c-Cre^+^.TN3 (*n* = 5) and CD11c-Cre^–^.TN3 (*n* = 4) males at 7 MPI. (**I**) Correlation between numbers of cDCs and mRNA level of STAT1 within kidneys from CD11c-Cre^+^.TN3 and CD11c-Cre^–^.TN3 males. Statistical analysis is done by 1-way ANOVA with Tukey’s multiple-comparison test (**A**), 2-way ANOVA with Holm-Šídák multiple-comparison test (**B**), 2-tailed Mann-Whitney *U* test (**C**–**F** and **H**), and Spearman’s correlation (**G** and **I**). *P < 0.05, **P < 0.01.

Since restoration of ROS specifically in CD68^+^ cells limits pDC accumulation and IFN signature, fully protecting *Ncf1^m1j/m1j^*.MN^Tg^ mice from PIL exacerbation, we hypothesized that the protection is, at least partially, because of intrinsic ROS in pDCs. To further verify the role of NOX2-derived ROS produced by pDCs in lupus, we used the B6N.Q. CD11c-Cre^+^.TN3 (CD11c-Cre^+^.TN3) strain, which expresses functional NCF1 protein only in CD11c^+^ cells. Unlike human pDCs, which are CD11c^–^, mouse pDCs express CD11c at a low level (21). As expected, the capacity to make an ROS response was partially restored in conventional DCs (cDCs) and pDCs of the CD11c-Cre^+^.TN3 strain. Neither CD11c-Cre^+^.TN3 nor CD11c-Cre^–^.TN3 mice displayed oxidative burst in neutrophils or macrophages/monocytes (Figure 4A).

We then injected pristane to induce experimental lupus in CD11c-Cre^+^.TN3 mice. Mice with restored ROS in DCs developed lower levels of autoantibodies against nucleosomes and Smith/ribonucleoprotein (Sm/RNP) during the development of the disease, while there was no difference in the levels of anti-dsDNA IgG (Figure 4B). Kidney injury was less severe in CD11c-Cre^+^.TN3 mice compared with CD11c-Cre^–^.TN3 mice, as indicated by lower urinary protein levels at 6 MPI (Figure 4C). Furthermore, lower expression of STAT1 and other ISGs was found in kidneys from CD11c-Cre^+^.TN3 mice (Figure 4, D and E). Consistently, we observed milder renal infiltration of pDCs in CD11c-Cre^+^.TN3 male mice than in their sex-matched Cre^–^ littermates, which correlated with *Stat1* expression (Figure 4, F and G). In contrast, kidney-infiltrating cDCs exhibited neither difference in cell counts nor a correlation with *Stat1*, further indicating that ROS-sufficient pDCs are the major regulatory cells ameliorating the disease (Figure 4, H and I).

In conclusion, our results show ROS produced by DCs ameliorate lupus and lupus nephritis through regulation of pDC accumulation and IFN responses.

### ROS produced by DCs prevent spontaneous development of lupus in Ncf1-mutant mice with Yaa.

Having found that ROS produced by DCs ameliorate PIL, we next asked if DC-derived ROS protect mice from ROS deficiency–induced disease exacerbation as seen in *Ncf1^m1j/m1j^*.MN^Tg^. For this purpose, we established a mouse strain carrying both the *Ncf1^m1j^* mutation and the Y-linked autoimmune accelerating locus (*Yaa*). *Yaa* is a region translocated from the X chromosome onto the Y chromosome, resulting in spontaneous lupus in the male mice with lupus-prone backgrounds (18). The duplicated *Tlr7* gene in the *Yaa* and the resultant overexpression of TLR7 lead to lupus-like disease phenotypes, demonstrating the important role of TLR7-induced type I IFNs as the causative pathway for lupus (18). It is known that pDCs potently produce type I IFNs in response to ligation of TLR7, which can be activated by RNA-containing ICs and are associated with B cell activation and lupus development (22). Therefore, we considered *Yaa* mice as a useful tool to investigate the protective role of ROS in pDCs in lupus.

**Figure 5:**
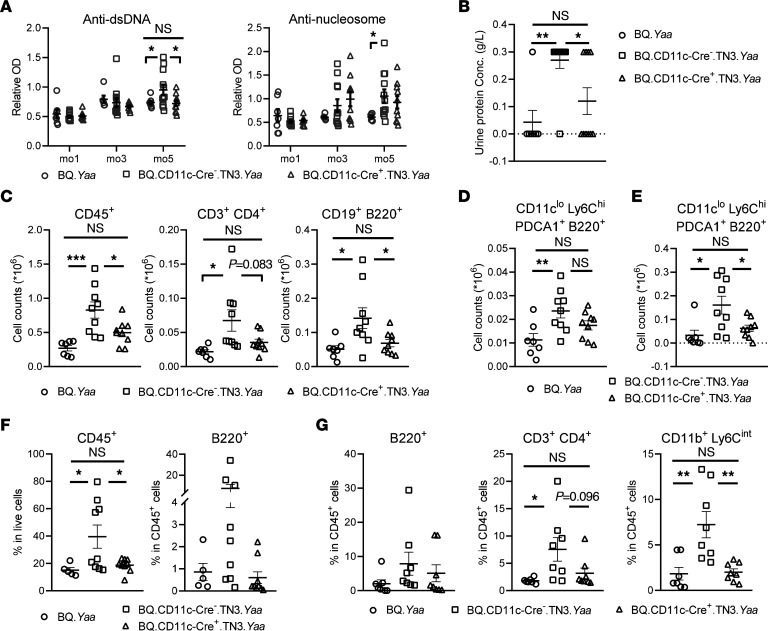
ROS produced by DCs prevent spontaneous development of lupus in *Ncf1*-mutant mice with *Yaa*. (**A**) Serum levels of autoantibodies against dsDNA and nucleosomes in BQ.*Yaa* (*n* = 8), BQ.CD11c-Cre^–^.TN3.*Yaa* (*n* = 12), and BQ.CD11c-Cre^+^.TN3.*Yaa* mice (*n* = 11) at different ages. (**B**) Urinary protein concentrations in 5-month-old BQ.*Yaa* (*n* = 7), BQ.CD11c-Cre^–^.TN3.*Yaa* (*n* = 10), and BQ.CD11c-Cre^+^.TN3.*Yaa* (*n* = 10); (**C**) numbers of CD45^+^ cells, CD4^+^ T cells, and B cells within kidneys; (**D**) numbers of pDCs within kidneys, and (**E**) Numbers of pDCs within spleen from 5-month-old BQ.*Yaa* (*n* = 7), BQ.CD11c-Cre^–^.TN3.*Yaa* (*n* = 9), and BQ.CD11c-Cre^+^.TN3.*Yaa* mice (*n* = 10). (**F**) Frequency of CD45^+^ cells and B cells within salivary glands from 5-month-old BQ.*Yaa* (*n* = 5), BQ.CD11c-Cre^–^.TN3.*Yaa* (*n* = 9), and BQ.CD11c-Cre^+^.TN3.*Yaa* mice (*n* = 9). (**G**) Frequency of B cells, CD4^+^ T cells, and neutrophils within BAL fluids from 5-month-old BQ.*Yaa* (*n* = 7), BQ.CD11c-Cre^–^.TN3.*Yaa* (*n* = 8), and BQ.CD11c-Cre^+^.TN3.*Yaa* mice (*n* = 8). Statistical analysis is done by 2-way ANOVA with Tukey’s multiple-comparison test (**A**) and 1-way ANOVA with Tukey’s multiple-comparison test (**B**–**G**). *P < 0.05, **P < 0.01, ***P < 0.001.

We earlier observed spontaneous development of signs of lupus in Balb/c.*Ncf1^m1j^* mice; however, in mice with C57.black backgrounds such as B10.Q or C57BL/6, neither *Ncf1^m1j^* mutation nor *Yaa* alone spontaneously induces lupus-like disease (5, 23). We introduced *Yaa* into the CD11c-Cre.TN3 mouse line and observed the male mice over 5 months. BQ.CD11c-Cre^–^.TN3.*Yaa* males developed higher levels of autoantibodies against dsDNA and nucleosomes, as compared with BQ.*Yaa* (Figure 5A). Comparable levels of anti-dsDNA antibodies but not anti-nucleosome antibodies were found in the sera from BQ.CD11c-Cre^+^.TN3.*Yaa* and BQ.*Yaa* mice, suggesting that DC-specific ROS at least partially regulate autoantibody production (Figure 5A). In addition, we found similar urinary protein levels in the 5-month-old BQ.CD11c-Cre^+^.TN3.*Yaa* and BQ.*Yaa* male mice, significantly lower than in BQ.CD11c-Cre^–^.TN3.*Yaa* mice (Figure 5B). Consistently, we observed more total CD45^+^ cells, CD4^+^ T cells, and B cells in the kidneys of BQ.CD11c-Cre^–^.TN3.*Yaa* mice, while comparable numbers were found in BQ.CD11c-Cre^+^.TN3.*Yaa* and BQ.*Yaa* (Figure 5C). Although there was only a trend in total CD4^+^ T cells, the percentage of Foxp3^+^ Tregs was significantly higher in Cre^+^ mice than their Cre^–^ littermates (Supplemental Figure 3). These results indicate that DC-specific ROS protect ROS-deficient *Yaa* mice from kidney injuries and immune cell infiltration during lupus development.

Restoration of ROS in DCs limited pDC accumulation in kidneys and spleens in BQ.CD11c-Cre^+^.TN3.*Yaa* mice, to the same level as in BQ.*Yaa* (Figure 5, D and E). PDCs are also suggested to be involved in Sjögren’s syndrome (SS), another chronic autoimmune disorder closely related to SLE. SLE and SS share possible underlying pathogenic pathways, such as TLR activation and type I IFN signaling (24). Since lymphocytic infiltration in salivary glands is characteristic of SS and usually concomitantly occurs with lupus activity in other organs, we immunophenotyped the infiltrates within salivary glands from the 3 strains. We found more CD45^+^ cells and predominantly B cells in the salivary glands in BQ.CD11c-Cre^–^.TN3.*Yaa* mice, as compared with BQ.*Yaa* (Figure 5F). More importantly, comparable frequency of immune cells was found in BQ.CD11c-Cre^+^.TN3.*Yaa* and BQ.*Yaa* mice, indicating that DC-derived ROS fully prevent inflammation in salivary glands during lupus development in ROS-deficient *Yaa* mice.

Since lung involvement is common in SLE and has been reported in lupus-prone *Yaa* mice (25), we characterized leukocytes from bronchoalveolar lavage (BAL) fluids from BQ.*Yaa* and BQ.CD11c-Cre.TN3.*Yaa* males. Higher frequencies of B cells, CD4^+^ T cells, and neutrophils were found in BAL fluids from 5-month-old BQ.CD11c-Cre^–^.TN3.*Yaa* mice, as compared with the other 2 strains, while comparable frequency was seen between BQ.CD11c-Cre^+^.TN3.*Yaa* and BQ.*Yaa* mice (Figure 5G). This indicates that ROS produced by DCs prevent lupus-associated lung inflammation.

In conclusion, NCF1-dependent ROS deficiency drives spontaneous lupus development in mice carrying *Yaa*, reflected by autoantibodies against dsDNA and nucleosomes, kidney damage, and involvement of lungs and salivary glands. More importantly, ROS restoration specifically in DCs protect against lupus by controlling autoantibody production and preventing immune infiltration, especially pDC accumulation, in multiple organs.

### ROS regulate accumulation of pDCs and upregulation of ISGs at the initial stage of PIL.

**Figure 6:**
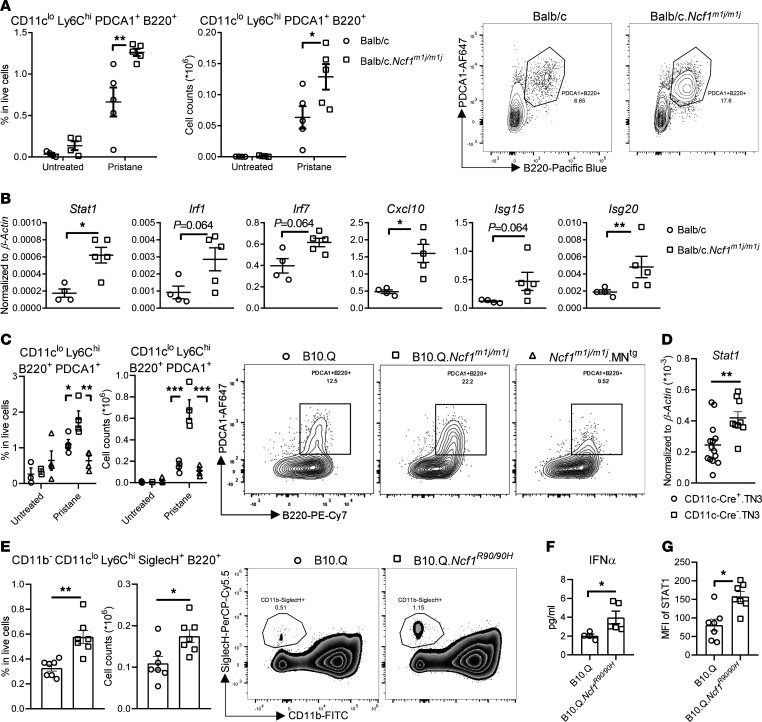
ROS regulate accumulation of pDCs and type I IFN responses at the initial stage of PIL. (**A**) Frequency and numbers of pDCs within peritoneal exudates (PECs) from naive and pristane-treated Balb/c and Balb/c.*Ncf1^m1j/m1j^* mice (*n* = 4–5) at day 3 after pristane injection. Representative flow cytometry plots from pristane-treated Balb/c and Balb/c.*Ncf1^m1j/m1j^* are presented. (**B**) Expression of ISGs within PECs from Balb/c and Balb/c.*Ncf1^m1j/m1j^* (*n* = 4–5) at day 3 after pristane injection. (**C**) Frequency and numbers of pDCs within PECs from naive and pristane-treated B10.Q, B10.Q.*Ncf1^m1j/m1j^*, and *Ncf1^m1j/m1j^*.MN^Tg^ (*n* = 3–4) at day 3 after pristane injection. Representative flow cytometry plots from pristane-treated B10.Q, B10.Q.*Ncf1^m1j/m1j^*, and *Ncf1^m1j/m1j^*.MN^Tg^ mice are presented. (**D**) Expression of *Stat1* within PECs from CD11c-Cre^+^.TN3 (*n* = 14) and CD11c-Cre^–^.TN3 mice (*n* = 9) at day 3 after pristane injection. (**E**) Frequency and numbers of pDCs within PECs from B10.Q (*n* = 7) and B10.Q.*Ncf1^R90/90H^* (*n* = 7) at day 3 after pristane injection. Representative flow cytometry plots are presented. (**F**) Level of IFN-α in peritoneal fluids from B10.Q (*n* = 4) and B10.Q.*Ncf1^R90/90H^* (*n* = 5) at day 3 after pristane injection. (**G**) Protein level of STAT1 within PECs from B10.Q (*n* = 7) and B10.Q.*Ncf1^R90/90H^* (*n* = 7) at day 3 after pristane injection. Statistical significance is determined by 2-way ANOVA with Holm-Šídák multiple-comparison test (**A**), 2-tailed Mann-Whitney *U* test (**B** and **D**–**G**), and 1-way ANOVA with Tukey’s multiple comparison test (**C**). *P < 0.05, **P < 0.01, ***P < 0.001.

Using *Ncf1^m1j/m1j^*, *Ncf1^m1j/m1j^*.MN^Tg^, CD11c-Cre^+^.TN3, and CD11c-Cre^+^.TN3.*Yaa* mice, we demonstrated accumulation of pDCs in various target organs during PIL and spontaneous lupus development, which is regulated by NOX2-derived ROS. Next, to investigate when the regulatory effects occur, we immunophenotyped the cells in the peritoneum, where initial activation of the immune system starts in the PIL model. By analyzing the pristane-induced peritoneal pathology at the early stage (day 3 after pristane injection), we found that Balb/c.*Ncf1^m1j/m1j^* mice exhibited dramatic accumulation of various immune cells, such as B cells, NK cells, macrophages, and most strikingly, pDCs within peritoneal exudates (Figure 6A and Supplemental Figure 4A). In concert with the number of pDCs, higher expression of ISGs was found in the peritoneum of Balb/c.*Ncf1^m1j/m1j^* mice than WT mice, suggesting a stronger ISG and inflammatory response (Figure 6B). However, *Ncf1^m1j/m1j^*.MN^Tg^ and B10.Q mice displayed comparable frequency and numbers of pDCs in the peritoneum at day 3 after pristane injection (Figure 6C), and CD11c-Cre^+^.TN3 mice showed significantly lower *Stat1* expression than their Cre^–^ littermates (Figure 6D). These results showed that CD68^+^CD11c^+^ cell–derived ROS regulation of pDC accumulation and type I IFN responses occur at the initial stage of lupus.

### Insufficient ROS production in Ncf1^R90/90H^ mice carrying the human NCF1-339 SNP potentiates pDC responses.

To verify that the alteration in pDCs and ISGs observed in *Ncf1^m1j/m1j^* mice is relevant to humans with *NCF1*-339, we phenotyped the peritoneal cells from B10.Q.*Ncf1^R90/90H^* mice at day 3 after pristane injection. This strain carries the human *NCF1*-339 SNP on an allele of *Ncf1*, leading to an amino acid exchange from arginine to histidine (from R90 to 90H), which results in reduced NOX2-derived ROS production (Supplemental Figure 4B). We found higher frequency and numbers of pDCs in the peritoneum of *Ncf1^R90/90H^* mice compared with WT mice (Figure 6E). Unlike in *Ncf1^m1j/m1j^* mice, no difference in any other immune cell type was found in *Ncf1^R90/90H^*, which further indicates the importance of pDCs (Supplemental Figure 4C). Moreover, *Ncf1^R90/90H^* mice displayed higher levels of IFN-α in the peritoneal fluids, which was consistent with our observation in CD11c-Cre.TN3 strains (Supplemental Figure 5). Furthermore, we found higher expression of STAT1 in the peritoneal cells of *Ncf1^R90/90H^* mice than their WT littermates (Figure 6, F and G). Our results demonstrate that moderate decrease in ROS production in *Ncf1^R90/90H^* mice specifically promotes pDC accumulation and resultant type I IFN activation at an early stage of lupus.

### Generation of pDCs is enhanced via AKT/mTOR pathway in ROS-deficient mice.

**Figure 7:**
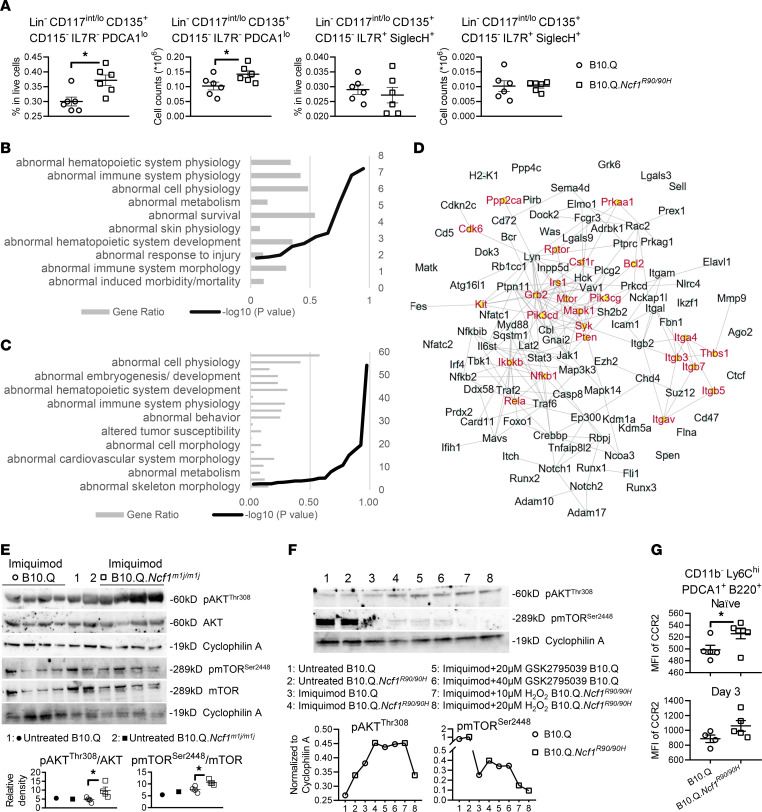
Generation of pDCs is enhanced via AKT/mTOR pathway in ROS-deficient mice. (**A**) Frequency and numbers of pre-pDCs within BM from B10.Q and B10.Q.*Ncf1^R90/90H^* (*n* = 6) at day 3 after pristane injection. (**B** and **C**) Monarch mammalian phenotype enrichment analysis of (**B**) proteomic expression profile and (**C**) PISA of BM-derived pDCs from B10.Q and B10.Q.*Ncf1^m1j/m1j^* mice upon 20-hour stimulation by IFN-α. (**D**) Protein-protein interaction networks (STRING) of the matching proteins in the PISA data set that are relevant to “abnormal hematopoietic system morphology/development” in Mammalian Phenotype Ontology. The proteins related to “PI3K-Akt-mTOR signaling pathway” in WikiPathways are illustrated in red. (**E**) Expression of p-AKT^Thr308^ and p-mTOR^Ser2448^ in BM SiglecH^+^ cells from B10.Q and B10.Q.*Ncf1^m1j/m1j^* mice upon 15-minute stimulation by imiquimod. The densitometric ratio of the phosphorylated protein to the total protein is calculated. (**F**) Expression of p-AKT^Thr308^ and p-mTOR^Ser2448^ in splenocytes from B10.Q and B10.Q.*Ncf1^R90/90H^* with or without GSK2795039 or H_2_O_2_ upon 15-minute stimulation by imiquimod. Quantification of bands normalized to cyclophilin A is presented. (**G**) Expression of CCR2 in BM pDCs from naive B10.Q (*n* = 5), naive B10.Q.*Ncf1^R90/90H^* (*n* = 6), pristane-treated B10.Q (*n* = 4), and pristane-treated B10.Q.*Ncf1^R90/90H^* (*n* = 5) mice at day 3 after pristane injection. Statistical analysis is done by 2-tailed Mann-Whitney *U* test (**A**, **E**, and **G**) and 2-tailed Student’s *t* test (**B** and **C**). *P < 0.05.

Since ROS regulation of pDC accumulation occurs already at the initial stage of PIL, we hypothesized that ROS deficiency promotes generation of pDCs in BM. It is known that pDCs originate from common DC progenitors (CDPs) and common lymphoid progenitors (26, 27). We found more CDP-derived pre-pDCs in *Ncf1^R90/90H^* BM at day 3 after pristane injection, suggesting that the higher number of pDCs in ROS-deficient mice is at least partially due to enhanced pDC production (Figure 7A). However, at naive status, the number of pre-pDCs was comparable between WT and *Ncf1^R90/90H^* mice, indicating that the difference in pDC progenitors is regulated (Supplemental Figure 6).

To further characterize how ROS affects pDC generation, we compared the proteomic landscape of WT and *Ncf1^m1j/m1j^* BM-derived pDCs after in vitro stimulation by IFN-α, which autocrinally supports a broad range of pDC activities, including development, differentiation, maturation, and survival (28, 29). BM cells were sorted by magnetic beads with anti-B220 mAb, and the resultant population was a mixture of mature pDCs and CCR9^–^ pDC precursors, making it suitable for studying pDC development (30). Besides deep expression profiling, to capture the differences in other aspects such as protein-protein interactions and posttranslational modifications, we also assessed the protein solubility and/or thermal stability with the Proteome Integral Solubility Alteration (PISA) assay (31). In fact, differentially expressed proteins in *Ncf1^m1j/m1j^* pDCs were mainly enriched in processes related to hematopoiesis/myelopoiesis, immune responses, and redox activity (Figure 7B). The PISA assay also revealed protein enrichment in hematopoietic development (Figure 7C). To identify the underlying pathways, we analyzed the protein-protein interactions among the proteins clustered in the hematopoiesis process in the PISA assay with STRING database and found the PI3K/AKT/mTOR signaling pathway to be the major one involved in ROS-regulated pDC development (Figure 7D). To validate the findings, we isolated BM SiglecH^+^ cells, which consist of 50% pDC precursors and 20% mature pDCs (Supplemental Figure 7), and stimulated them with imiquimod, a TLR7 agonist, since TLR7 ligation has been shown to promote mTOR-dependent emergency myelopoiesis, leading to massive myeloid cell and DC expansion (32). We observed higher phosphorylation of mTOR and its upstream signal AKT in *Ncf1^m1j/m1j^* cells, as compared with ROS-sufficient WT cells (Figure 7E). To confirm that upregulation of AKT/mTOR pathway is ROS dependent, we abolished NOX2-derived ROS production in imiquimod-treated B10.Q splenocytes by a specific NOX2 inhibitor, GSK2795039 (GSK), and supplied *Ncf1* *^R90/90H^* splenocytes with H_2_O_2_ (33). At 20 μM GSK increased mTOR pathway in WT cells to a similar level as in *Ncf1^R90/90H^* cells, while 20 μM H_2_O_2_ reduced the signaling in *Ncf1^R90/90H^* (Figure 7F). Similar results were found in BM cells (Supplemental Figure 8A). These findings demonstrate that NCF1-dependent ROS control pDC generation through AKT/mTOR pathway.

Apart from myelopoiesis, enhanced cellular migration may also contribute to pDC accumulation during lupus. It is known that TLR7 activation induces pDC migration to the inflammatory sites via the CCL2/CCR2 axis (34). CCR2 is an inducible inflammatory chemokine receptor involved in leukocyte traffic in both humans and murine lupus nephritis (35, 36). It also promotes pDCs’ egress from BM at steady state (37). We observed significantly higher CCR2 expression on mature pDCs in BM from *Ncf1^R90/90H^* naive mice, as compared with WT mice, and a similar trend at day 3 after pristane injection (Figure 7G). Decreased CCR2 expression after rapamycin treatment indicated its association with the mTOR pathway (Supplemental Figure 8B). We also measured other molecules known to be involved in pDC migration under certain conditions, including CXCR3, CXCR4, CCR5, CCR6, CCR7, β_1_/β_2_ integrins, CD62L, and endothelial adhesion molecules ICAM-1 and VCAM-1 (38), on BM and peritoneal pDCs from naive and pristane-treated mice. None of these molecules showed different expression levels between WT and *Ncf1^R90/90H^* pDCs (Supplemental Figure 9).

Taken together, low ROS production due to NCF1 deficiency enhances AKT/mTOR-dependent pDC generation and promotes pDC migration via CCR2, which may explain the accumulation of pDCs during lupus.

### Hyperactive ROS-deficient pDCs exacerbate PIL.

We next investigated whether intrinsic ROS regulate the functionality of pDCs. Since pDCs are rare cells, for subsequent experiments, we mostly used pDCs derived from BM.

**Figure 8:**
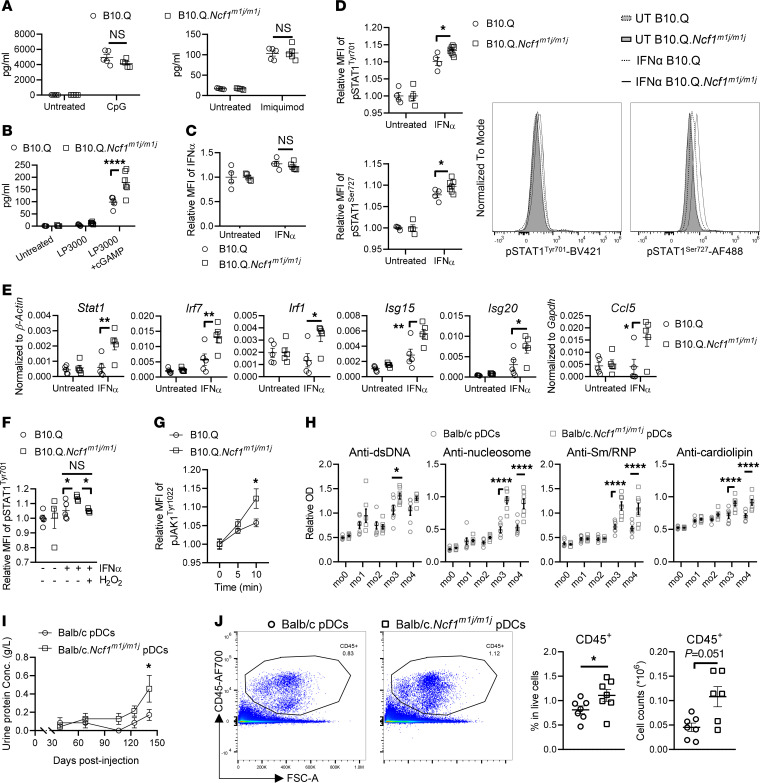
ROS-deficient pDCs with hyperactivated STING/IFN-α/JAK1/STAT1 cascade exacerbate PIL. (**A** and **B**) IFN-α production by BM-derived pDCs from B10.Q and B10.Q.*Ncf1^m1j/m1j^* mice upon 24-hour stimulation by (**A**) TLR7/9 agonists and (**B**) cGAMP. (**C**) IFN-α production by BM-derived pDCs from B10.Q and B10.Q.*Ncf1^m1j/m1j^* mice upon 20-hour stimulation by IFN-α. (**D**) P-STAT1 in BM-derived pDCs from B10.Q and B10.Q.*Ncf1^m1j/m1j^* mice upon 30-minute stimulation by IFN-α. Representative histograms are presented. (**E**) Expression of ISGs in BM-derived pDCs from B10.Q and B10.Q.*Ncf1^m1j/m1j^* mice upon 4-hour stimulation by IFN-α. (**F**) P-STAT1 in BM-derived pDCs from B10.Q and B10.Q.*Ncf1^m1j/m1j^* mice with or without H_2_O_2_ upon 30-minute stimulation by IFN-α. (**G**) P-JAK1 in BM-derived pDCs from B10.Q and B10.Q.*Ncf1^m1j/m1j^* mice upon stimulation by IFN-α for indicated times. (**H**) Serum levels of autoantibodies against dsDNA, nucleosomes, Sm/RNP, and cardiolipin and (**I**) urinary protein concentrations in Balb/c mice receiving Balb/c pDCs and Balb/c mice receiving Balb/c.*Ncf1^m1j/m1j^* pDCs (*n* = 7) at different time points after pristane injection. (**J**) Frequency and numbers of CD45^+^ cells within kidneys from Balb/c mice receiving Balb/c pDCs and Balb/c mice receiving Balb/c.*Ncf1^m1j/m1j^* pDCs (*n* = 7) at 5 MPI. Representative plots are presented. Two-way ANOVA with Holm-Šídák multiple-comparison test (**A**–**E** and **G**–**I**), 1-way ANOVA with Tukey’s multiple-comparison test (**F**), and 2-tailed Mann-Whitney *U* test (**J**). *P < 0.05, **P < 0.01, ****P < 0.0001.

It is known that pDCs potently produce type I IFNs in response to ligation of nucleic acid–sensing pattern recognition receptors (PRRs), such as TLR7 and TLR9, which have been implicated in lupus development (22). We stimulated BM-derived pDCs from WT and *Ncf1^m1j/m1j^* mice with the TLR7 agonist imiquimod and TLR9 agonist CpG ODN2395. Both induced comparable level of IFN-α production by ROS-sufficient and -deficient pDCs (Figure 8A). In addition, activation of the STING pathway has recently been shown to trigger IFN-α production in pDCs from SLE patients (39), although its involvement in experimental lupus models is under debate (40). We treated WT and *Ncf1^m1j/m1j^* pDCs with the STING agonist 2′3′-cyclic guanosine monophosphate (2′3′-cGAMP) and found higher secretion of IFN-α by *Ncf1^m1j/m1j^* cells (Figure 8B). The autocrine/paracrine signaling where IFN-α further induces production of itself revealed no difference between the strains (Figure 8C).

Next, we assessed the STAT1 pathway, which is downstream of IFN-α stimulation. As a member of ISGs, STAT1 has been reported to be upregulated at mRNA and protein levels in whole blood from *Ncf1^m1j/m1j^* mice and *NCF1*-339–carrying patients (5, 7), yet its activity has not been clearly addressed in NCF1-deficient cells. We found higher phosphorylation of STAT1 in *Ncf1^m1j/m1j^* pDCs than WT pDCs (Figure 8D), and consequently, higher transcription of ISGs (Figure 8E). These results indicate that ROS-deficient pDCs are hyperresponsive to IFN-α through STAT1 signaling, which forms a vicious cycle of type I IFN responses.

To verify that activation of STAT1 is regulated by ROS, we stimulated pDCs with or without H_2_O_2_ supplement. *Ncf1^m1j/m1j^* pDCs, in the presence of H_2_O_2_, displayed a comparable level of p-STAT1 to WT pDCs after IFN-α stimulation, while p-STAT1 in ROS-deficient pDCs cultured without H_2_O_2_ was higher than in WT pDCs (Figure 8F). We therefore conclude that ROS-deficient pDCs display hyperactivation of IFN-α/STAT1 signaling, which can be limited by ROS.

JAK1, one of the tyrosine kinases giving upstream signals to STAT1, is known to be sensitive to ROS (41). We hypothesized that ROS regulate STAT1 by modifying JAK1 activity. To test the hypothesis, we measured JAK1 phosphorylation in pDCs upon stimulation with IFN-α and observed higher p-JAK1 in ROS-deficient *Ncf1^m1j/m1j^* pDCs, as compared with WT pDCs (Figure 8G). Blocking JAK1 activity with a JAK inhibitor, baricitinib, decreased the levels of p-STAT1 in ROS-deficient pDCs in vitro (Supplemental Figure 10A). Moreover, oral administration of baricitinib ameliorated spontaneous lupus in *Ncf1^m1j/m1j^*.*Yaa* mice (Supplemental Figure 10, B–D). These results suggest that insufficient ROS regulation of JAK1 is at least partially responsible for stronger STAT1 activation in pDCs and exacerbation of lupus in *Ncf1^m1j/m1j^* mice.

Having found that ROS-deficient pDCs displayed increased IFN-α production and type I IFN responses via the STING and JAK1/STAT1 pathways, we further investigated if these hyperreactive cells exacerbate lupus. We transferred Balb/c WT and Balb/c.*Ncf1^m1j/m1j^* pDCs to pristane-treated Balb/c WT mice and followed them for 5 months. We found higher levels of autoantibodies against dsDNA, nucleosomes, Sm/RNP, and cardiolipin in the mice receiving ROS-deficient pDCs, as compared with those receiving ROS-sufficient pDCs (Figure 8H). They also developed more severe lupus nephritis, as indicated by higher urinary protein concentration (Figure 8I). Consistently, the mice receiving *Ncf1^m1j/m1j^* pDCs exhibited more inflammatory cell infiltration, predominantly macrophages and B cells, in the kidneys (Figure 8J and Supplemental Figure 11).

Taken together, our results show that ROS-deficient pDCs are functionally hyperactive and promote lupus development.

## Discussion

The association between NOX2-derived ROS deficiency and autoimmunity was first observed in CGD patients who developed a chronic granulomatous disease with symptoms of colitis, lupus, and arthritis (42). Originally based on positional cloning of a gene polymorphism underlying arthritis in animals (43), genetic studies later identified mutations in NOX2 subunits to be linked to various human autoimmune diseases, including SLE (4). The sequence of the human *NCF1* locus is not yet fully clarified due to a large number of variable copies; however, exon sequencing revealed several SNPs (6). Recently, one of the amino acid–replacing SNPs, *NCF1*-339, leading to an *NCF1* allele with lower ROS generation capacity, was discovered in 2 independent studies based on multi-ancestry cohorts to be strongly associated with SLE (7, 8). To investigate the role of NOX2-derived ROS in lupus, *Ncf1^m1j/m1j^* mice carrying a spontaneous mutation in the *Ncf1* gene served as a scientific tool. Reduced formation of NETs was observed in pristane-injected *Ncf1^m1j/m1j^* mice, which indicates that NOX2-derived ROS protect against lupus through NETs (11). Similar results have been obtained using samples from patients with SLE and *NCF1*-339 SNP carriers (14, 44). Another mechanism was suggested by Hahn et al. that impaired phagocytosis of apoptotic cells by *Ncf1^m1j/m1j^* monocytes and neutrophils may activate the immune system with undigested apoptotic materials in *Ncf1^m1j/m1j^* mice (14). Consistent with this hypothesis, Geng and colleagues showed in *NCF1*-339 knockin mice that lower ROS production leads to reduced phagosomal acidification, thereby impairing proteolysis and efferocytosis of macrophages, which promotes follicular T helper 2 responses (9). Similarly, Meng et al. showed that ROS deficiency in mice carrying *NCF1*-339 leads to lower endosomal pH and enhanced TLR signaling in pDCs, which in turn results in increased type I IFN secretion and exacerbation of imiquimod-induced lupus (12).

In the current study, by using a set of cell-specific Cre-deleter and transgenic mouse strains, we systematically screened and were able to identify pDCs as the major immune cell type responsible for lupus exacerbation due to NCF1 deficiency. NCF1-dependent ROS specifically restored in these cells protected mice from lupus in both PIL and a *Yaa*-accelerating spontaneous model. Apart from previously proposed mechanisms contributing to lupus susceptibility in NCF1-deficient mice, we suggest another mechanism that intrinsic ROS produced by pDCs limit AKT/mTOR-dependent pDC generation, CCR2-mediated tissue accumulation, and pDC hyperactivation through type I IFN signaling, thereby protecting against lupus development.

Abnormally high serum level of IFN-α has long been documented in studies of SLE (45). Its correlation to lupus severity and activity makes it a marker of immune activation in the disease. PDCs, identified as major IFN-α–producing cells, were therefore carefully investigated in SLE patients (46). Increased frequency of pDCs was reported in PBMCs from lupus patients after stimulation by virus or combination of GM-CSF and IFN-α/γ in vitro (46). However, the number of circulating pDCs was lower in SLE patients (47). The finding of pDCs in tissues, such as skin and lymph nodes, from patients with SLE may explain the decreased number in PBMCs (48). Similarly, more pDCs were found in lymphoid tissues from lupus-prone mice (49). Although ablation of pDCs in various models has proved their critical role in lupus development, few studies addressed mechanisms behind their accumulation in the disease (50–54).

In this study, by comparing the frequency and numbers of pDCs in ROS-deficient and -sufficient mice at different stages of lupus, we found that pDCs accumulated in multiple organs and correlated with local type I IFN signature as well as disease severity. This is regulated by pDC-intrinsic ROS and starts already at the initial stage of PIL, concomitant with enhanced pDC-poiesis. A recent study revealed transcriptional reprogramming in hematopoietic cells skewing toward the myeloid lineage in lupus mice and SLE patients (55). In line with this, we showed more pDCs developed from myeloid progenitors than those from lymphoid progenitors, which was, more importantly, promoted by ROS deficiency due to the *Ncf1*-339 SNP. Redox regulation of hematopoiesis is complex. A low level of ROS is required for the maintenance of quiescence status and the differentiation potential of hematopoietic cells, while an increase in intrinsic ROS production is necessary for activation of differentiation-related pathways, including AKT/mTOR (56). ROS have been reported to exert inhibitory or stimulatory effects in both hematopoietic process and the AKT pathway, depending on the sources of ROS, types of cells, and disease contexts (57–59). Here, we show that NCF1-dependent ROS at a physiological level limit development of pDCs in lupus through inhibition of the AKT/mTOR signaling. Consistently, the RNA-Seq analysis by Meng et al. identified the PI3K/AKT pathway when comparing ROS-deficient *NCF1*-339 SNP–carrying pDCs with ROS-sufficient WT pDCs after TLR7 stimulation.

Furthermore, we observed CCR2 upregulation on NCF1-deficient pDCs, which may favor their migration and accumulation during lupus. Although the underlying mechanism is unclear, this may be mediated by the AKT pathway since it has been suggested that an AKT-dependent positive feedback loop between β-catenin and CCR2 sustains high expression of CCR2 (60). Nevertheless, very little is known regarding the role of ROS in expression of CCR2 and other chemokine receptors. On the other hand, the involvement of ROS in chemokine production has been demonstrated (61). RNA profiling data based on pDCs from mice carrying the human *NCF1*-339 SNP revealed upregulation of various chemokines including CCL3, CCL4, CXCL9, CXCL10, and CXCL11 via the NF-κB signaling pathway (12).

It is well established that pDCs produce IFN-α upon stimulation by nucleic acid–containing materials from various sources, including apoptotic/necrotic cells, pathogens, and NETs (62), effects most likely triggered through Fc-γ receptors (FcγRs) and PRRs. Apoptotic cell–derived materials, in the form of ICs with autoantibodies, have been shown to trigger IFN-α production in pDCs via FcγRIIa and contribute to lupus development (63). Upon FcγR-mediated uptake, nucleic acids are sensed by the endosomal PRRs, TLR7 and TLR9, leading to type I IFN production through IRF7 signal cascade (64). The pathogenic role of TLR7 and the regulatory role of TLR9 in pDCs have been demonstrated in spontaneous murine models of lupus and PIL (65, 66). Recently, stimulation of another PRR, cyclic GMP-AMP synthase, by cytosolic DNA, was found to trigger IFN-α production in pDCs from patients with SLE and differentiation of pDCs in PIL, through activation of the STING pathway (39, 67). Moreover, pDCs can produce IFN-α through an autocrine/paracrine loop (68). In the present study, the difference between IFN-α secretion in ROS-sufficient and -deficient pDCs was seen after treatment with STING agonists, not TLR7/9 agonists or IFN-α. On the contrary, Meng et al. found that pDCs from *NCF1*-339–carrying mice secreted higher levels of IFN-α upon TLR7/9 activation (12). As Meng’s study demonstrated, *NCF1*-339 enhanced TLR signaling by lowering the pH values in late endosomes/lysosomes where TLRs locate. Since NOX2-derived ROS are known to be important in lysosome biogenesis (69), we suggest that the inconsistency between our and Meng’s data can be due to molecular differences between *Ncf1^m1j^* mutation and *NCF1*-339 SNP. *Ncf1^m1j^* entirely blocks NOX2-derived ROS production because of aberrant NCF1 protein expression, which results in reduced lysosome formation, thereby counterbalancing the effects of endosomal acidification on TLR-dependent IFN-α secretion. In contrast, *NCF1*-339 only slightly impairs NCF1 translocation to endosomes, leading to low endosomal pH, which affects TLR cleavage and the TLR signaling (12). Nevertheless, both studies demonstrate high IFN-α production in ROS-deficient pDCs, consistent with the observation that pDCs with oxidative stress–related gene signatures were too exhausted to produce IFN-α in patients with SLE (47).

In the current study, we demonstrated that ROS deficiency enhances the IFN-α/JAK1/STAT1 pathway in *Ncf1^m1j/m1j^* pDCs. This is in agreement with the previous finding that the genes related to the JAK/STAT pathway were enriched in the RNA-Seq analysis comparing R848-stimulated pDCs with and without the *NCF1*-339 SNP (12). Of importance, here we showed that hyperactivated IFN-α/JAK1/STAT1 signaling, in concert with increased STING-dependent IFN-α production in *Ncf1^m1j/m1j^* pDCs, acting as a positive feedback loop, augments type I IFN responses, which may be crucial to lupus exacerbation driven by ROS deficiency. This notion is strongly supported by the experiment showing that transfer of *Ncf1^m1j/m1j^* pDCs aggravates PIL.

Taken together, in this study, for the first time to our knowledge, we demonstrated that NOX2-derived ROS in pDCs protect against lupus development. This opens possibilities for treatment of SLE at least in patients carrying relevant genetic variants.

## Methods

### Animals.

Balb/cByJ and C57BL/6NJ were originally from The Jackson Laboratory. B6N.Q (C57/B6N.Q/rhd) and B10.Q (C57/B10N.Q/rhd) have been fully backcrossed into B6N and B10 genomes but with an MHC region from DBA/1, and maintained in our lab (Medical Inflammation Research). A mutation in the *Ncf1* gene (*m1j*) leads to a truncated NCF1 protein and thereby a nonfunctional NOX2 complex (13). The derived *Ncf1*-mutant mouse strains on different backgrounds were designated as Balb/c.*Ncf1^m1j/m1j^*, B6N.Q.*Ncf1^m1j/m1j^*, and B10.Q.*Ncf1^m1j/m1j^*, respectively. *Ncf1^R90H^* was established with a knockin R90H variant in the *Ncf1* gene by CRISPR/Cas9 in B6N embryonic stem cells (Shanghai Model Organisms Center) and backcrossed to B10.Q. We denote R90H variant expressed heterozygously as B10.Q.*Ncf1^R90/90H^*. The transgenic strain B10.Q.*Ncf1^m1j/m1j^*.MN^Tg^ (denoted as *Ncf1^m1j/m1j^*.MN^Tg^) express functional NCF1 predominantly in macrophages/monocytes using the human CD68 promoter (70). The transgenic strains CD11c-Cre^Tg^ (stock 018967) and Mrp8-Cre^Tg^ (stock 021614) from The Jackson Laboratory were crossed with a targeted *Ncf1* knockin strain lacking NOX2-derived ROS (B6N.Q.*Ncf1*^TN3/TN3^) (71). In vivo allelic conversion of *Ncf1* by Cre recombinase led to functional NCF1 expression in DCs (B6N.Q.CD11c-Cre^+^.TN3, denoted as CD11c-Cre^+^.TN3) and neutrophils (B6N.Q.Mrp8-Cre^+^.TN3, denoted as Mrp8-Cre^+^.TN3). These 2 transgenes were used when expressed heterozygously. Cre^+^.TN3 were compared with Cre^–^.TN3 as littermate controls in the experiments. B6.SB-*Yaa*/J (stock 000483) from The Jackson Laboratory has been fully backcrossed onto B10.Q, denoted as B10.Q.*Yaa*. The *Yaa*-carrying strain with fully functional NCF1 (BQ.*Yaa*), with NCF1 restored only in DCs (BQ.CD11c-Cre^+^.TN3.*Yaa*), or without NCF1 restored (BQ.CD11c-Cre^–^.TN3.*Yaa*) was obtained by crossing B6N.Q.CD11c-Cre^+^.TN3 with B10.Q.*Yaa*. Mice were housed under specific pathogen–free (FELASA II) conditions in individual ventilated cages with wood shaving bedding in a climate-controlled environment with a 12-hour light/12-hour dark cycle. Animal experiments were performed in a controlled way balanced for age and sex; the genotype of the mice was hidden from the investigators, following the Animal Research: Reporting of In Vivo Experiments guidelines.

### PIL.

Mice were injected intraperitoneally with a single dose of 500 μL pristane (MilliporeSigma, P2870) and followed up for 5–7 months. PIL arthritis was monitored using a macroscopic scoring system as described before (10). Briefly, 5 points were given to each visibly inflamed (erythema and swelling) ankle or wrist and 1 point to each inflamed toe. Histopathological evaluation on the ankle joints collected at the endpoint (5 MPI) by H&E staining was also performed (72).

### Detection of autoantibodies and cytokines.

For detection of murine autoantibodies by ELISA, microtiter plates were coated with 10 μg/mL nucleosomes isolated from apoptotic cells as described previously (73), 50 μg/mL cardiolipin (MilliporeSigma, C1649), and 10 U/mL Sm/RNP (GenWay Biotech, GWB-A1CECE) or precoated with 20 μg/mL poly-l-lysine (MilliporeSigma, P2658) before adding 20 μg/mL calf thymus DNA (MilliporeSigma, D3664). Sera were 1:50 or 1:25 diluted in PBS/2% FBS (Thermo Fisher Scientific, 26140079). Bound IgG was detected with HRP-conjugated goat anti-mouse IgG(H+L) (Southern Biotech, 1031-05) at 1:4,000 dilution, followed by substrate solution (Roche, 11112422001). Results were expressed as fold-changes relative to a designated positive sample. IFN-α level in peritoneal lavages and cell culture supernatants was quantified by cytometric bead array (BioLegend, 740633).

### Analysis of renal injury.

Proteinuria was determined with semiquantitative urine testing strips (Uristix, 2857) using midstream urine. For immunofluorescence, cryosections of kidneys were examined by a Zeiss LSM800 Microscope or an Olympus IX73 Inverted Microscope after being stained with Alexa Fluor 488–conjugated goat anti–mouse IgG specific for Fcγ fragment (Jackson ImmunoResearch, 115-545-071). Deposits were semiquantitatively graded on a scale of 0–3 (0 = none; 1 = weak; 2 = moderate; 3 = strong) according to the intensity by 2 individual scorers in a masked manner. At least 10 glomeruli/section were analyzed.

### Flow cytometry.

To prepare single-cell suspensions, the perfused kidneys were digested with 1 mg/mL collagenase (Roche, 11088866001) and 0.1 mg/mL DNase I (Roche, 10104159001) in a 37°C water bath for 45 minutes. Cells from kidney tissues, salivary glands, BAL fluids, PECs, peripheral blood, spleen, BM, or BM-derived cells were counted using Sysmex KX-21 Hematology Analyzer.

After a homemade anti–mouse CD16/CD32 FcR block (clone: 2.4G2), the cells were incubated with LIVE/DEAD Fixable Near-IR Dead Cell Stain Kit (Thermo Fisher Scientific, L10119) and labeled with the following antibodies: anti-CD11c (clone: HL3, PE or PE-Cyanine7; BD Biosciences); anti-Ly6C (clone: HK1.4, FITC or BV605 or APC; BioLegend); anti-PDCA1 (clone: 927, Alexa Fluor 647 or FITC; BioLegend); anti-B220 (clone: RA3-6B2, BV605 or FITC or Pacific Blue or PE-Cyanine7 or PE; BD Biosciences); anti-CD11b (clone: M1/70, Pacific Blue or FITC; BioLegend); anti-CD45 (clone: 30-F11, AF700; BioLegend); anti-CD68 (clone: FA-11, Alexa Fluor 647; BioLegend); anti–IFN-α (clone: RMMA-1, FITC; pbl assay science); anti-STAT1 (clone: 1/Stat1, PE; BD Biosciences); anti–p-STAT1^Tyr701^ (clone: 4a, BV421 or PerCP/Cy5.5; BD Biosciences); anti–p-STAT1^Ser727^ (clone: K51-856, Alexa Fluor 488; BD Biosciences); anti-JAK1 (clone: 413104, Alexa Fluor 488; R&D Systems); anti–p-JAK1^Tyr1022^ (polyclonal, Alexa Fluor 647; Bioss Antibodies); anti-F4/80 (clone: BM8, APC or PerCP/Cy5.5; BioLegend); anti–H-2, I-A/I-E (clone: M5/114.15.2, Pacific Blue; BioLegend); anti–H-2, I-Aq (clone: KH116, Alexa Fluor 647; BioLegend); anti-SiglecH (clone: 551, APC or PE or PerCP/Cy5.5; BioLegend); anti-Ly6G (clone: 1A8, PE; BioLegend); anti-CD19 (clone: 6D5, PE-Cyanine7; BioLegend); anti-CD3ε (clone: 500A2, PerCP/Cy5.5; BioLegend); anti-CD115 (clone: AFS98, PE; BioLegend); anti-CD127 (clone: A7R34, BV605; BioLegend); biotinylated anti-CD135 (clone: A2F10; BioLegend); anti-CCR9 (clone: 9B1, PE; BioLegend); anti-CCR2 (clone: 475301, APC; R&D Systems); and anti–c-Kit (clone: 2B8, APC-R700; BD Biosciences). Flow cytometric analysis was performed as previously described (5).

### Analysis of gene and protein expression.

Frozen kidneys were homogenized in TRIzol Reagent (Thermo Fisher Scientific, 15596026). According to manufacturer’s instructions, RNA from kidney homogenates and PECs was extracted with PureLink RNA Mini Kit (Thermo Fisher Scientific, 12183018A), and the concentration was determined by Thermo Fisher Scientific NanoDrop 2000 Spectrophotometers. CDNA was synthesized with iScript cDNA Synthesis Kit (Bio-Rad, 1708891). Gene expression was detected by Bio-Rad CFX96 Touch Real-Time PCR Detection System with iQ SYBR Green Supermix (Bio-Rad, 1708882). Results were expressed as Ct values normalized to β*-Actin* or *Gapdh*. Protein from kidney homogenates and cells was extracted and quantified by Pierce BCA Protein Assay Kit (Thermo Fisher Scientific, 23225). Samples were subjected to electrophoresis and transferred to nitrocellulose membranes (Thermo Fisher Scientific, LC2001). Membranes were incubated with antibodies against STAT1 (clone: D1K9Y, Cell Signaling Technology), p-STAT1^Tyr701^ (clone: D4A7, Cell Signaling Technology), p-AKT^Thr308^ (clone: 244F9, Cell Signaling Technology), p-mTOR^Ser2448^ (polyclonal, Cell Signaling Technology), and cyclophilin A (Thermo Fisher Scientific, PA1-025) at 1:1,000 dilution, followed by incubation with HRP-conjugated goat anti–rabbit IgG (Thermo Fisher Scientific, 31460) at 1:20,000 dilution. The Bio-Rad ChemiDoc XRS+ Gel imaging system was used for chemiluminescence detection. All the full Western blots are shown in the online supplemental material.

### ROS measurement.

Intracellular and extracellular oxidative burst were measured according to the protocols described before (7). Briefly, for intracellular ROS detection, cells were incubated with 3 μM DHR (Thermo Fisher Scientific, D23806) for 10 minutes after staining of cell surface markers, followed by stimulation with 200 ng/mL PMA (MilliporeSigma, P8139) for 20 minutes at 37°C. Fluorescence emitted from DHR after oxidation by ROS was detected by flow cytometry, and data were presented as relative geometric MFI of DHR in comparison with solvent control. For extracellular ROS measurement, neutrophils were magnetically isolated from BM with biotinylated anti-Ly6G mAb (BioLegend, clone: 1A8) according to manufacturer’s instructions from Miltenyi Biotec. The isolated cells were then diluted in Hanks’ balanced salt solution (HBSS; Thermo Fisher Scientific, 14025050) at a concentration of 10 × 10^6^ cells/mL, and 50 μL/well was added to a white 96-well plate (Thermo Fisher Scientific, 136101). Cells were mixed with 50 μL/well of HBSS with 350 μg/mL isoluminol (MilliporeSigma, A8264), 3.5 U/mL HRP (MilliporeSigma, P8250), and 200 ng/mL PMA. Luminescence signal was detected immediately and followed at 37°C using BioTek Synergy 2.

### In vitro pDC development.

BM cells were flushed from 2 femurs and 2 tibias per mouse. After lysis of red blood cells with ammonium-chloride-potassium buffer for 1 minute at room temperature, cells were plated at 2 × 10^6^ cells/mL in RPMI complete media containing 200 ng/mL FLT3L (BioLegend, 550704) for pDC differentiation. Cells were collected on day 7–9.

### Enrichment of pDCs and pDC precursors.

PDCs derived from BM were magnetically isolated (purity 70%–80%) with biotinylated anti-Ly6C mAb (BioLegend, clone: HK1.4). PDCs’ precursors were enriched (purity 50%) from Flt3L-cultured BM cells with biotinylated anti-B220 mAb (BD Biosciences, clone: RA3-6B2) or from primary BM cells with biotinylated anti-SiglecH mAb (Miltenyi Biotec, clone: 551.3D3) followed by magnetic separation.

### Stimulation of pDCs and pDC precursors.

Sorted pDCs as described above were cultured at a density of 1 × 10^5^ cells/well in RPMI complete medium in a 96-well, round-bottom plate (Falcon, Corning, 353077). For measurement of IFN-α production, pDCs were stimulated with 1 μM CpG ODN2395 (InvivoGen, tlrl-2395), 1 μg/mL imiquimod (InvivoGen, tlrl-imqs), 4 μg/mL 2′3′-cGAMP (InvivoGen, tlrl-nacga23-02) with Lipofectamine 3000 Transfection Reagent (LP3000, Thermo Fisher Scientific, L3000001), or 500 U/mL IFN-α (BioLegend, 752804) for 20 hours or 24 hours. A total of 3 μg/mL Brefeldin A (MilliporeSigma, B7651) was added for the last 3 hours of incubation with IFN-α. For detection of p-STAT1, p-JAK1, and ISG expression, pDCs were stimulated with 1,000 U/mL IFN-α for indicated times. To supply *Ncf1^m1j/m1j^* pDCs with H_2_O_2_, 10 μM H_2_O_2_ was added to the wells prior to IFN-α stimulation. Phosphorylation of JAK1 and STAT1 was shown as the relative MFI of p-JAK1 and p-STAT1 to the untreated controls. For detection of p-mTOR and -AKT, enriched pDC precursors were stimulated with 2 μg/mL imiquimod for 15 minutes.

### Stimulation of splenocytes.

A total of 6 × 10^6^ cells/well splenocytes from B10.Q and B10.Q.*Ncf1^R90/90H^* naive mice were stimulated with 2 μg/mL imiquimod for 15 minutes in 24-well cell culture plates (Sarstedt, 83.3922). To inhibit NOX2-dependent ROS production in WT splenocytes, 20 μM or 40 μM GSK was added before imiquimod stimulation, while 10 μM or 20 μM H_2_O_2_ was added to *Ncf1^R90/90H^* splenocytes for ROS supplementation.

### Transfer of pDCs.

PDCs were derived from BM from Balb/c or Balb/c.*Ncf1^m1j/m1j^* mice as described above. Balb/c mice were administrated intraperitoneally with the first injection of 5 × 10^5^ pDCs at 24 hours after pristane injection. Afterward, 5 × 10^5^ pDCs were injected every week intraperitoneally another 3 times. At 10 weeks after pristane injection, the mice were boosted with 5 × 10^5^ pDCs intravenously.

### PISA assay.

PDCs were derived from BM and isolated with biotinylated anti-B220 mAb as described above. A total of 5 × 10^6^ pDCs were plated in 6-well cell culture plates (Sarstedt, 83.3920) and stimulated with 500 U/mL IFN-α for 20 hours. For PISA assay, we followed our previously published protocol (31). After treatment, pDCs were collected by centrifugation at 350*g* and 4°C for 5 minutes, washed twice with PBS, and then resuspended in approximately 300 μL PBS. The cell suspension from each replicate was aliquoted into 10 in 96-well plates and heated in an Eppendorf gradient thermocycler (Mastercycler X50) in the temperature range of 48°C–59°C for 3 minutes. Samples were cooled for 3 minutes at room temperature and afterward snap-frozen with liquid nitrogen. The aliquots from each replicate were pooled and subjected to 4 more freeze-thaw cycles. The samples were then transferred into polycarbonate thick-wall tubes and centrifuged at 100,000*g* and 4°C for 20 minutes. The soluble protein fraction was then collected, protein concentration was measured using BCA assay, and 25 μg of each sample was taken. Dithiothreitol (DTT) was added to a final concentration of 10 mM, and samples were incubated for 45 minutes at room temperature. Subsequently, iodoacetamide was added to a final concentration of 50 mM, and samples were incubated at room temperature for 1 hour in the dark. The reaction was quenched by adding an additional 10 mM of DTT. Proteins were precipitated by methanol/chloroform and resuspended in 20 mM 4-(2-Hydroxyethyl)-1-piperazinepropanesulfonic acid (EPPS) buffer pH 8.5 with 8 M urea. Urea was diluted to 4 M by adding 20 mM EPPS. Lysyl endopeptidase (LysC; Wako) was added at a 1:75 w/w ratio at room temperature overnight. Samples were diluted with 20 mM EPPS to a final urea concentration of 1 M, and trypsin was added at a 1:75 w/w ratio, followed by 6-hour incubation at room temperature.

For profiling expression, we followed our previously published protocol (74). Cells were lysed with 1% SDS, 8 M urea, and Tris buffer pH 8.0 plus protease inhibitor. The cell lysates were subjected to 1 minute of sonication using Branson probe sonicator with 3-second on/off pulses and 30% amplitude. The concentration of proteins was measured using BCA assay, and the volume corresponding to 25 μg of protein was transferred from each sample to new tubes. The rest of the protocol is similar to above.

Acetonitrile was added to a final concentration of 20% and 200 μg Tandem Mass Tag (TMT) reagents was added to each PISA or expression sample, followed by incubation for 2 hours at room temperature. The reaction was quenched by addition of 0.5% hydroxylamine. PISA and expression samples were pooled, acidified by trifluoroacetic acid, cleaned using Sep-Pak cartridges (Waters), and dried using DNA 120 SpeedVac Concentrator (Thermo Fisher Scientific). The TMT multiplexing scheme is shown in Table 1.

The pooled samples were resuspended in 20 mM ammonium hydroxide and separated into 96 fractions on an XBrigde BEH C18 2.1 × 150 mm column (Waters; catalog 186003023), using a Dionex UltiMate 3000 2DLC system (Thermo Fisher Scientific) over a 48-minute gradient of 1%–63% B (B = 20 mM ammonium hydroxide in acetonitrile) in 3 steps (1–23.5% B in 42 minutes, 23.5%–54% B in 4 minutes, and then 54%–63% B in 2 minutes) at 200 μL/min flow. Fractions were then concatenated into 24 samples in sequential order (e.g., A1, C1, E1, and G1 on the 96-well plate were combined).

### Liquid chromatography-tandem mass spectrometry.

After drying, samples were dissolved in buffer A (0.1% formic acid and 2% acetonitrile in water). The samples were loaded onto a 50 cm EASY-Spray column (75 μm internal diameter, packed with PepMap C18, 2 μm beads, 100 Å pore size) connected to a nanoflow Dionex UltiMate 3000 UHPLC system (Thermo Fisher Scientific) and eluted in an organic solvent gradient increasing from 3 to 26% (B: 98% acetonitrile, 0.1% formic acid, 2% H_2_O) at a flow rate of 300 nL/min over 95 minutes. The eluent was ionized by electrospray, with molecular ions entering an Orbitrap Exploris mass spectrometers (Thermo Fisher Scientific) with the following settings: scan range of 375–1500, orbitrap resolution 120,000, MS automatic gain control target of 3 × 10^6^, MS maximum injection time 50 ms, higher energy collisional dissociation 33, MS2 resolution 45,000, MS2 AGC target of 2 × 10^5^, MS2 maximum injection time 120 ms, and isolation window 1.6.

### Data processing.

The raw liquid chromatography-tandem mass spectrometry data were analyzed by ProteomeDiscoverer 2.4. The Mascot search engine matched MS/MS data against the UniProt complete proteome database (human, version UP000005640_9606, 92,957 entries), unless otherwise specified. *Ncf1^m1j^* encoding sequence was also added to the above fasta file. Trypsin/P was selected as enzyme specificity. No more than 2 missed cleavages were allowed. A 1% false discovery rate was used as a filter at both protein and peptide levels. For all other parameters, the default settings were used. TMTpro16 (channels 1–10 were used in the current study) was used for peptide quantification. Cysteine carbamidomethylation was set as a fixed modification, while methionine oxidation was selected as a variable modification. The mass spectrometry proteomics data have been deposited to the ProteomeXchange Consortium via the PRIDE partner repository with the data set identifier PXD035964.

### Statistics.

For proteomics data, PISA experiments were performed in 2 replicates and expression experiments in 3 replicates. After removing the contaminants, protein intensities were normalized to the total protein intensity in each TMT channel. Subsequently, PISA data for each protein were normalized by corresponding expression channels (*Ncf1^m1j/m1j^* or WT littermates), and 6 ratios were obtained for *Ncf1^m1j/m1j^* and WT PISA samples. *P* values for the potential target proteins were calculated by *t* test based on fully normalized intensities between *Ncf1^m1j/m1j^* and WT littermates. Two-sided Student’s *t* test was applied to calculate *P* values. No peptides from the mutation site were quantified; therefore, for unbiased comparison, we removed the 2 peptides mapping to the sequence around this site in WT samples and recalculated the protein abundances in PISA and expression samples based on the other 12 peptides (there was only negligible change).

Statistical analysis was performed with GraphPad Prism version 8.0.1 by 2-tailed Mann-Whitney nonparametric test, Spearman’s correlation, or 1-way or 2-way ANOVA as specified in figure legends. *P* < 0.05 was considered significant. Data points represent measurement values of biological replicates. All results are shown as mean ± SEM.

### Study approval.

Stockholm Animal Ethics Committee, Stockholm, Sweden, 4516-2017, approved all the animal experiments.

## Author contributions

H Luo, VU, and RH conceived and designed the experiments. H Luo and VU performed most of the experiments and analyzed the data. AAS, H Lyu, MG, ÁV, and RAZ contributed to proteomic analysis. YL characterized the *Ncf1^R90H^* knockin strain. H Luo wrote the paper. VU and RH critically reviewed the manuscript. RH and VU jointly supervised the research.

## Supplementary Material

Supplemental data

## Figures and Tables

**Table 1:**
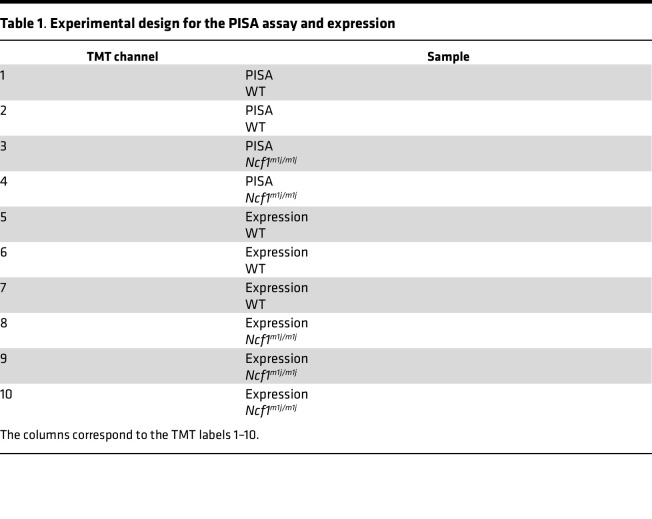
Experimental design for the PISA assay and expression

## Version 1 02/28/2023

In-Press Preview

## Version 2 04/10/2023

Electronic publication
