# Supplementary material for: NCF1-dependent production of ROS protects against lupus by regulating plasmacytoid dendritic cell development and functions
Source: JCI Insight. 2023 Apr 10;8(7):e164875. doi: 10.1172/jci.insight.164875 (PMC10132169; doi:10.1172/jci.insight.164875)

Supplemental Material for

NCF1-dependent production of ROS protects against lupus by regulating plasmacytoid dendritic cell development and functions

Huqiao Luo[1], Vilma Urbonaviciute[1], Amir Ata Saei[2,3], Hezheng Lyu[2], Massimiliano Gaetani[2,4,5], Ákos Végvári[2,6], Yanpeng Li[1,7], Roman A Zubarev[2], Rikard Holmdahl[1*]

*Corresponding author. Email: rikard.holmdahl@ki.se

Supplemental Figure 1

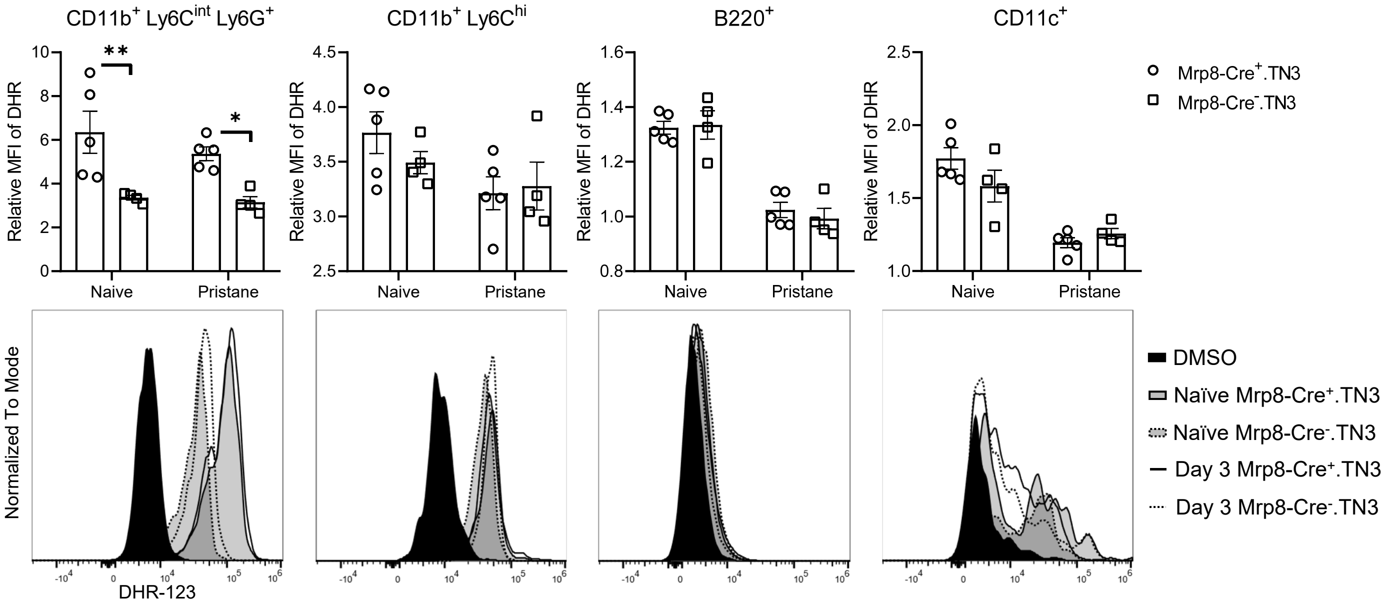

Supplemental Figure 1: ROS are specifically restored in neutrophils in Mrp8-Cre⁺.TN3 mice during PIL.

Oxidative burst in neutrophils, monocytes, B cells, and DCs within peripheral blood from naïve Mrp8-Cre⁺.TN3 (*n*=5) and Mrp8-Cre⁻.TN3 (*n*=4), and pristane-treated Mrp8-Cre⁺.TN3 (*n*=5) and Mrp8-Cre⁻.TN3 (*n*=4) mice at day 3 post pristane injection. Results are shown as mean ± SEM. Statistical significance is determined by two-way analysis of variance with Sidak's multiple comparison test and presented as asterisks (*p<0.05, **p<0.01). Representative histograms of ROS production in each cell type after PMA stimulation are presented. A DMSO-treated sample from a naïve Mrp8-Cre⁺.TN3 mouse is also shown to indicate the background.

Supplemental Figure 2

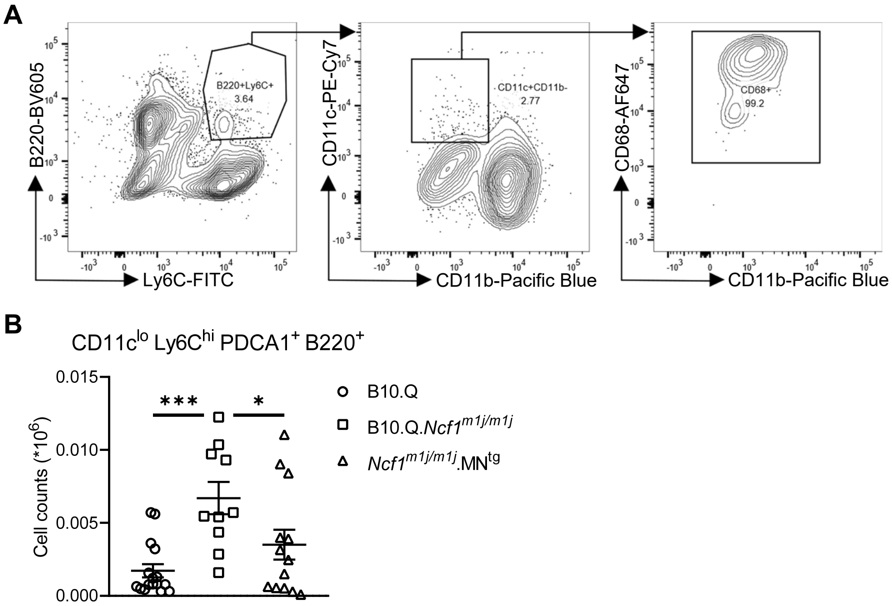

Supplemental Figure 2: NOX2-derived ROS produced by monocytes/macrophages and pDCs limit splenic pDC accumulation at the late stage of PIL.

(A) CD68 expression in pDCs. Sample data were pDCs ($CD11c^{+}$ $CD11b^{-}$ $Ly6C^{hi}$ $B220^{+}$) within spleen from a *Ncf1*$^{m1j/m1j}$.MN^{tg} mouse. **(B)** Numbers of pDCs within spleen from B10.Q (*n*=16), B10.Q.*Ncf1*$^{m1j/m1j}$ (*n*=10), and *Ncf1*$^{m1j/m1j}$.MN^{tg} (*n*=13) mice at 7-month post pristane injection. Data are pooled from two experiments. Results are shown as mean ± SEM. Statistical significance is determined by one-way analysis of variance with Tukey's multiple comparison test and presented as asterisks (*p<0.05, ***p<0.001).

Supplemental Figure 3

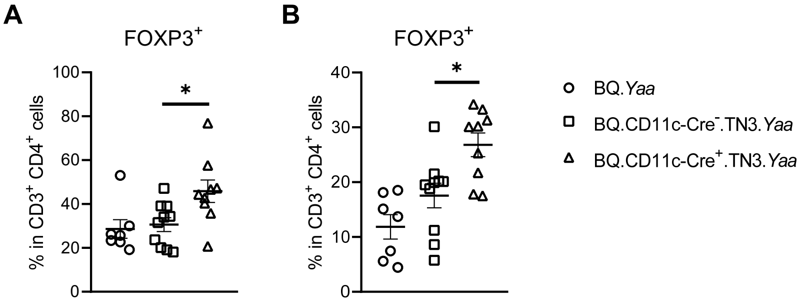

Supplemental Figure 3: BQ.CD11c-Cre⁺.TN3.*Yaa* mice exhibit higher percentage of Tregs within the CD4⁺ T cell population.

The percentage of FOXP3⁺ Tregs within the CD3⁺ CD4⁺ T cell population in the **(A)** kidneys and **(B)** spleen from 5-month-old BQ.*Yaa* (*n*=7), BQ.CD11c-Cre⁻.TN3.*Yaa* (*n*=9), and BQ.CD11c-Cre⁺.TN3.*Yaa* (*n*=10) mice. Results are shown as mean ± SEM. Statistical significance is determined by two-way analysis of variance with Tukey's multiple comparison test and presented as asterisks (*p<0.05).

Supplemental Figure 4

A

Cell populations	Frequency (% in live cells)	
	Balb/c.*Ncf1*$^{m1j/m1j}$	Balb/c
pDCs (CD11c^{lo} Ly6C^{hi} PDCA1^{+} B220^{+})	0.85 (**)	0.49
CD4^{+} T cells (CD3^{+} CD4^{+})	0.57	0.52
CD8^{+} T cells (CD3^{+} CD8^{+})	0.19	0.20
B cells (B220^{+})	3.76 (*)	2.56
NK cells (CD3^{-} CD49^{+})	1.70 (*)	0.79
Neutrophils (CD11b^{+} Ly6G^{+})	43.9 (*)	59.0
Monocytes (CD11b^{+} Ly6C^{hi})	10.9	8.82
Macrophages (CD11b^{+} Ly6C$^{low/-}$ F4/80^{+})	20.7	17.1

B

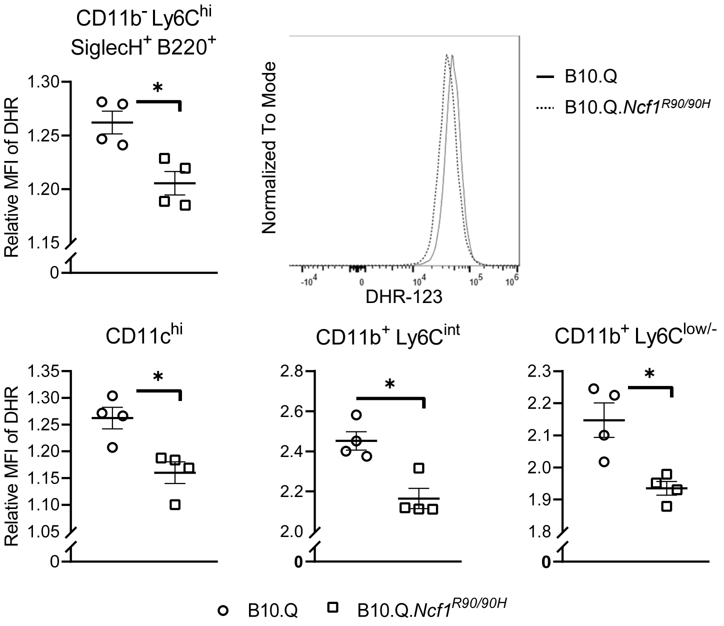

C

Cell populations	Frequency (% in live cells)	
	B10.Q.*Ncf1*$^{R90/90H}$	B10.Q
pDCs (CD11b^{-} CD11c^{lo} Ly6C^{hi} SiglecH^{+} B220^{+})	0.56 (**)	0.36
CD4^{+} T cells (CD3^{+} CD4^{+})	2.48	2.22
CD8^{+} T cells (CD3^{+} CD8^{+})	1.66	1.05
B cells (CD19^{+} B220^{+})	1.93	1.45
NK cells (CD3^{-} NK1.1^{+})	1.87	1.64
Neutrophils (CD11b^{+} Ly6G^{+})	28.6	38.3
Monocytes (CD11b^{+} Ly6C^{hi})	27.8	22.7
Macrophages (CD11b^{+} Ly6C$^{low/-}$)	17.0	15.7

Supplemental Figure 4: ROS-deficient mice exhibit peritoneal accumulation of pDCs at the initial stage of PIL.

(A) Mean frequency of indicated populations within PECs from Balb/c (*n*=4) and Balb/c.*Ncf1*$^{m1j/m1j}$ (*n*=5) mice at day 3 post pristane injection. **(B)** Oxidative burst in pDCs, cDCs, neutrophils, and macrophages within spleen from naïve B10.Q (*n*=4) and B10.Q.*Ncf1*$^{R90/90H}$ (*n*=4) mice. Representative histograms of ROS production in pDCs are presented. **(C)** Mean frequency of indicated populations within PECs from B10.Q (*n*=7) and B10.Q.*Ncf1*$^{R90/90H}$ (*n*=7) mice at day 3 post pristane injection. Results are shown as mean ± SEM. Statistical significance is determined by two-tailed Mann-Whitney U test and presented as asterisks (*p<0.05, **p<0.01).

Supplemental Figure 5

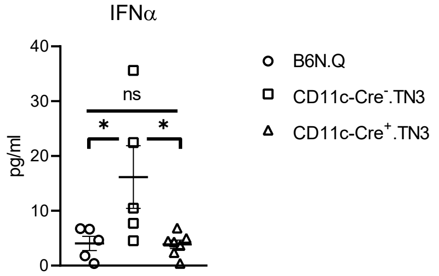

Supplemental Figure 5: Restoration of ROS in CD11c⁺ cells regulates IFNα secretion in the peritoneum at the initial stage of PIL.

Level of IFNα in peritoneal fluids from B6N.Q (*n*=5), CD11c-Cre⁻.TN3 (*n*=5), and CD11c-Cre⁺.TN3 (*n*=7) mice at day 3 post pristane injection. Results are shown as mean ± SEM. Statistical significance is determined by one-way analysis of variance with Tukey's multiple comparison test and presented as asterisks (ns: not significant, *$p<0.05$).

Supplemental Figure 6

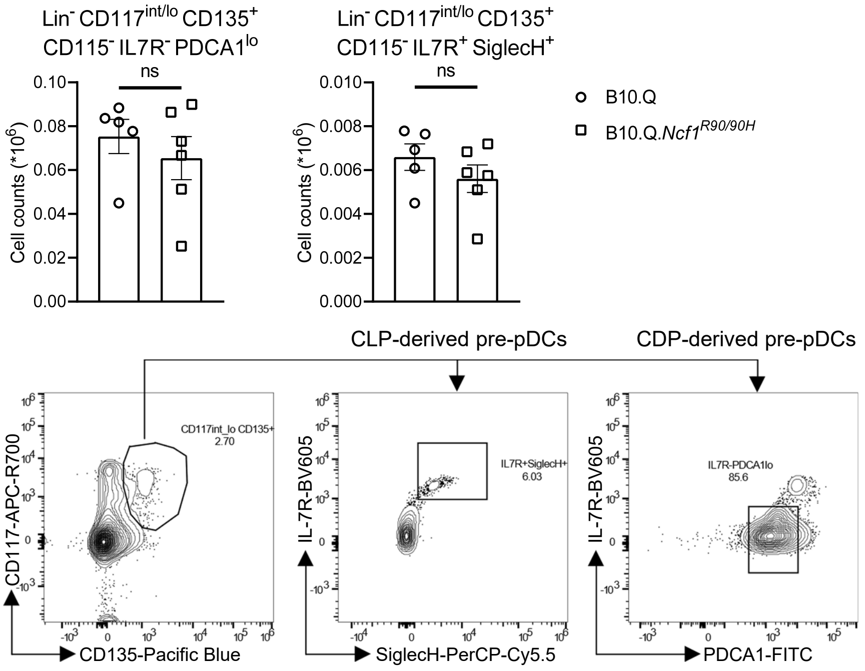

Supplemental Figure 6: ROS-deficient *Ncf1$^{R90/90H}$* mice and ROS-sufficient mice develop comparable numbers of pre-pDCs at naïve status.

Numbers of pre-pDCs within BM from naïve B10.Q (*n*=5) and B10.Q.*Ncf1$^{R90/90H}$* (*n*=6) mice. The gating strategies of CDP- and CLP-derived pre-pDCs are shown. CD117$^{int/lo}$ CD135^{+} population is pre-gated on B220^{-} Ly6C^{-} CD115^{-}.

Supplemental Figure 7

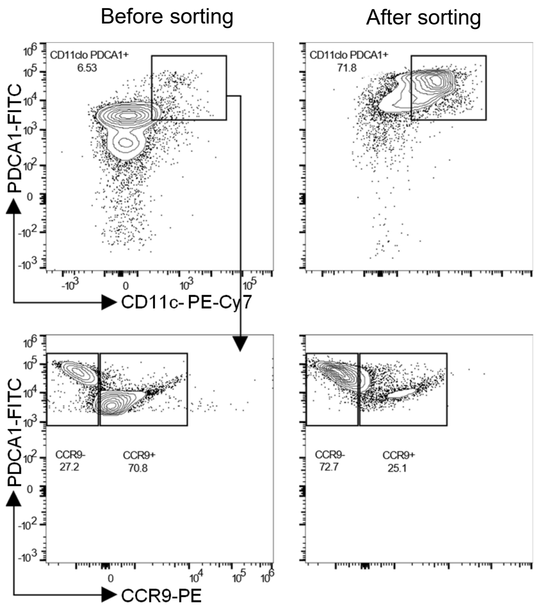

Supplemental Figure 7: BM SiglecH^{+} population contains mature pDCs and pDC precursors.

Frequency of mature pDCs (CD11c^{lo} PDCA1^{+} CCR9^{+}) and pDC precursors (CD11c^{lo} PDCA1^{+} CCR9^{-}) within BM before and after magnetic isolation using anti-SiglecH antibodies. Sample data were unsorted and sorted B10.Q BM cells.

Supplemental Figure 8

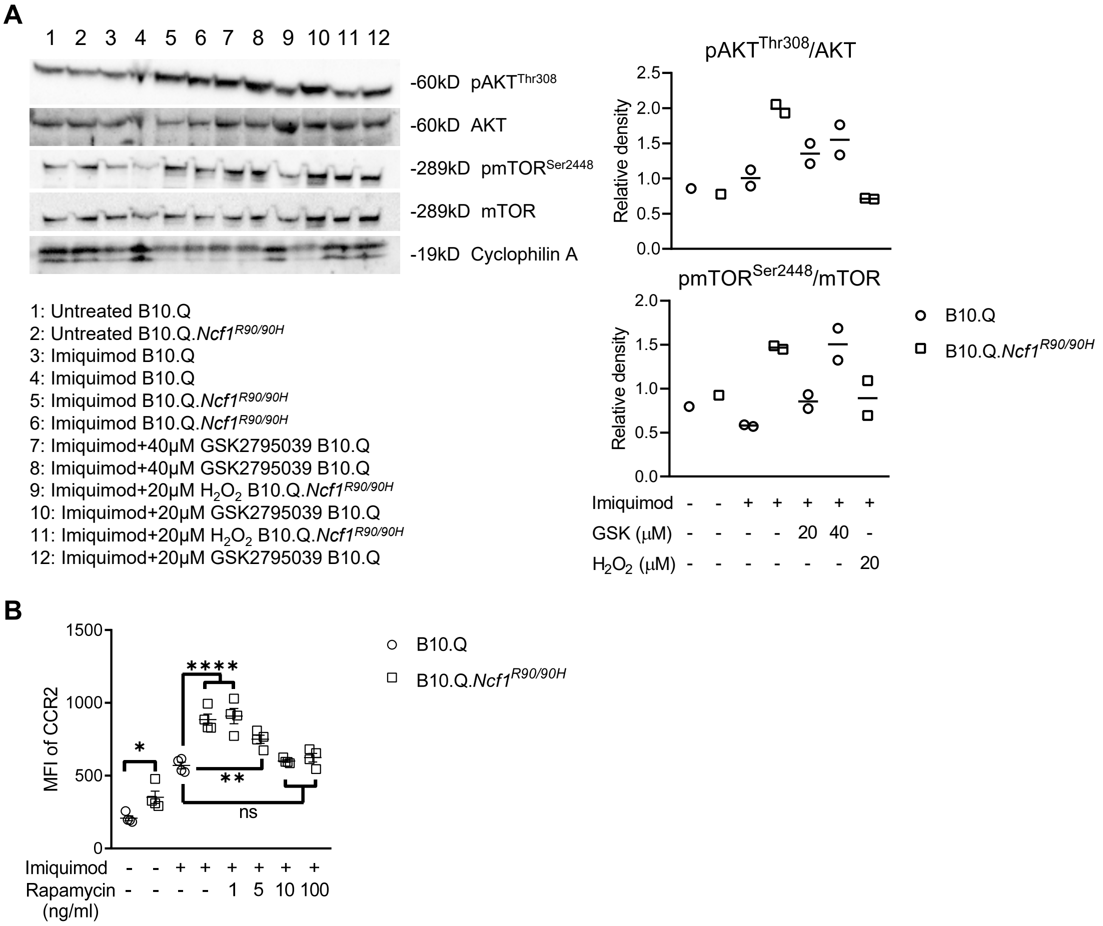

Supplemental Figure 8: ROS deficiency in *Ncf1^R90/90H^* mice results in upregulation of the AKT/mTOR pathway and CCR2 expression.

(A) Expression of pAKT^{Thr308} and pmTOR^{Ser2448} in BM cells from B10.Q and B10.Q.*Ncf1*$^{R90/90H}$ mice with or without GSK2795039 or H_2O_2 upon 15min stimulation by imiquimod. The densitometric ratio of the phosphorylated protein to the total protein is presented. **(B)** Expression of CCR2 in BM pDCs from B10.Q and B10.Q.*Ncf1*$^{R90/90H}$ mice with or without the mTOR inhibitor rapamycin upon 24h stimulation by 2μg/ml imiquimod. Results are shown as mean in (A) and mean ± SEM in (B). Statistical significance is determined by one-way analysis of variance with Dunnett's multiple comparison test and presented as asterisks (ns: not significant, *p<0.05, **p<0.01, ****p<0.0001).

Supplemental Figure 9

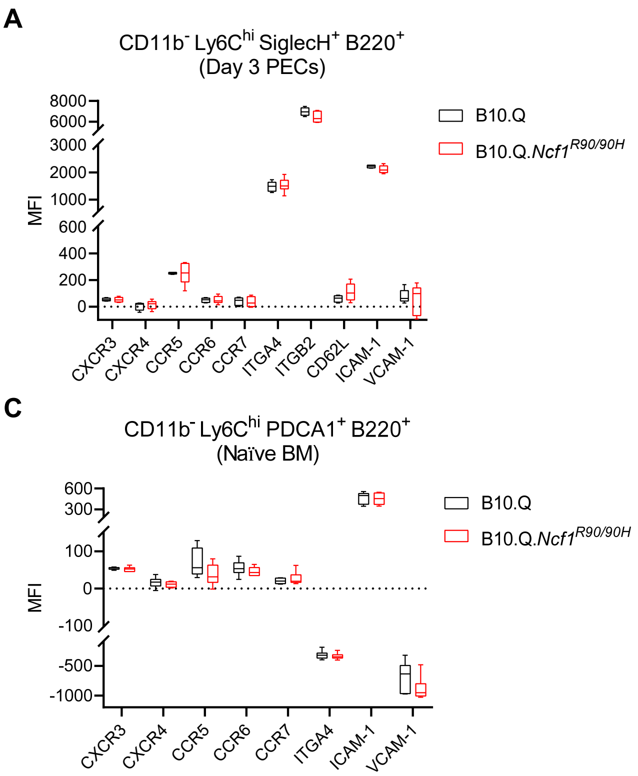

Supplemental Figure 9: ROS-deficient *Ncf1$^{R90/90H}$* mice and ROS-sufficient mice express comparable levels of migration receptors on pDCs.

(A) Expression of CXCR3, CXCR4, CCR5, CCR6, CCR7, ITGA4, ITGB2, CD62L, ICAM-1, and VCAM-1 on pDCs within PECs from B10.Q (*n*=9) and B10.Q.*Ncf1$^{R90/90H}$* (*n*=10) mice at day 3 post pristane injection. Data are pooled from two experiments. **(B)** Expression of CXCR3, CXCR4, CCR5, CCR6, CCR7, ITGA4, CD62L, ICAM-1, and VCAM-1 on pDCs within BM from B10.Q (*n*=5) and B10.Q.*Ncf1$^{R90/90H}$* (*n*=5) mice at day 3 post pristane injection. **(C)** Expression of CXCR3, CXCR4, CCR5, CCR6, CCR7, ITGA4, CD62L, ICAM-1, and VCAM-1 on pDCs within BM from naïve B10.Q (*n*=5) and B10.Q.*Ncf1$^{R90/90H}$* (*n*=5) mice. Box-and-whisker plots show medians, 25th/75th percentiles, and minimum/maximum values of MFI. Statistical significance is determined by two-tailed Mann-Whitney U test (not significant).

Supplemental Figure 10

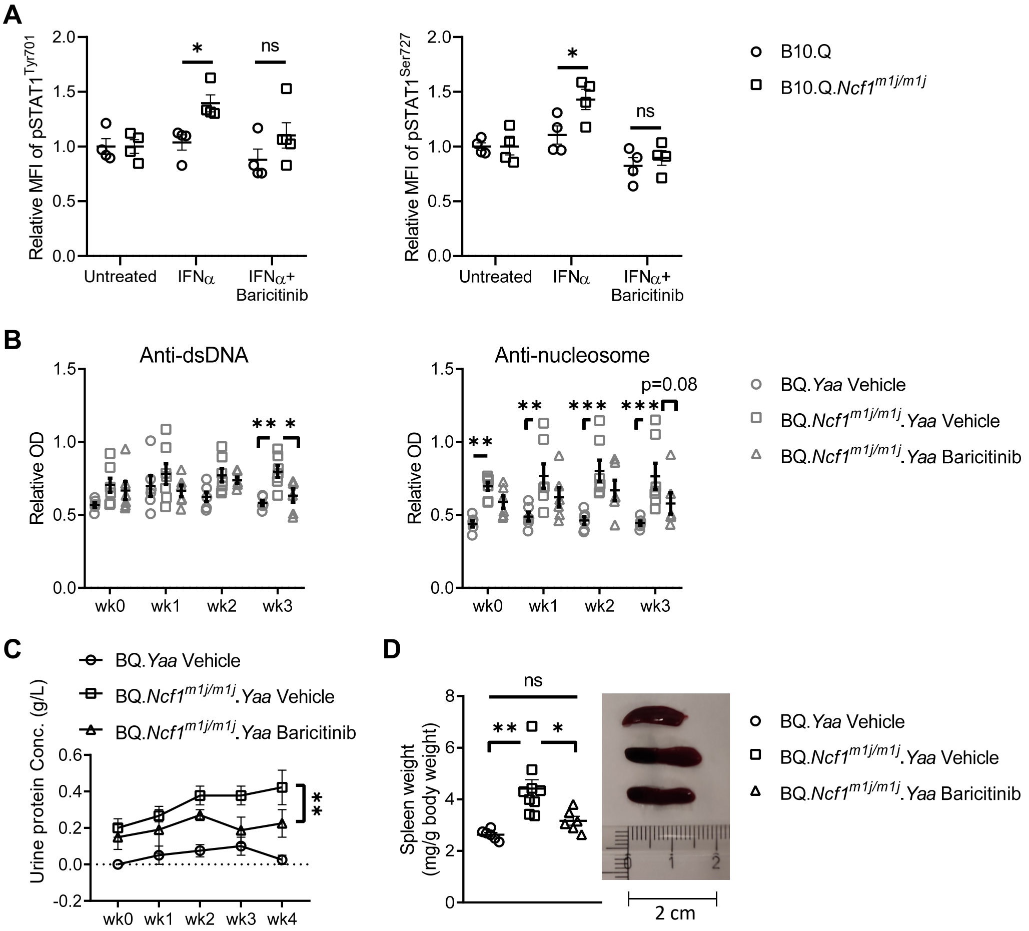

Supplemental Figure 10: The JAK1/2 inhibitor baricitinib reduces STAT1 phosphorylation in ROS-deficient pDCs and ameliorates spontaneous lupus in *Ncf1*-mutant mice with *Yaa* locus.

(A) Phospho-STAT1 in BM-derived pDCs from B10.Q and B10.Q.*Ncf1*$^{m1j/m1j}$ mice with or without 0.5μM baricitinib treatment upon 30min stimulation by 1000U/ml IFNα. Baricitinib was added to the cells 1h prior to IFNα stimulation. **(B-D)** The experiment was performed in the *Yaa*-carrying strains with functional NCF1 (BQ.*Yaa*) and mutant NCF1 (BQ.*Ncf1*$^{m1j/m1j}$.*Yaa*). Baricitinib was orally administrated to the BQ.*Ncf1*$^{m1j/m1j}$.*Yaa* mice once daily at 20mg/kg in 0.5% carboxymethylcellulose (Sigma-Aldrich, #C5678) from 12 weeks of age for a week. The control groups were BQ.*Yaa* mice and BQ.*Ncf1*$^{m1j/m1j}$.*Yaa* mice receiving 0.5% carboxymethylcellulose (vehicle). The mice were followed for 4 weeks after the first day of gavage. **(B)** Serum levels of autoantibodies against dsDNA and nucleosomes, **(C)** Urinary protein concentrations, and **(D)** Spleen weight at 4-week after the first day of gavage in BQ.*Yaa* mice treated with vehicle (*n*=6), BQ.*Ncf1*$^{m1j/m1j}$.*Yaa* mice treated with vehicle (*n*=9), and BQ.*Ncf1*$^{m1j/m1j}$.*Yaa* mice treated with baricitinib (*n*=6). Results are shown as mean ± SEM. Statistical analysis is done by two-way analysis of variance with Sidak's multiple comparison test in (A), two-way analysis of variance with Tukey's multiple comparison test in (B-C), and one-way analysis of variance with Tukey's multiple comparison test in (D). Significance is presented as asterisks (ns: not significant, *p<0.05, **p<0.01, ***p<0.001). Representative images of spleens of the three groups of mice were shown next to the spleen weight plot.

Supplemental Figure 11

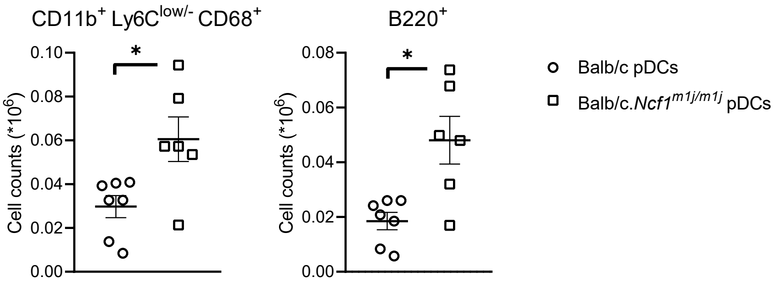

Supplemental Figure 11: ROS-deficient pDCs promote renal infiltration of macrophages and B cells in PIL.

Numbers of macrophages and B cells within kidneys from Balb/c mice receiving Balb/c pDCs (*n*=7) and Balb/c mice receiving Balb/c.*Ncf1*$^{m1j/m1j}$ pDCs (*n*=7) at 5-month post pristane injection. Results are shown as mean ± SEM. Statistical significance is determined by two-tailed Mann-Whitney U test and presented as asterisks (*p<0.05).

Western Blot Raw Data

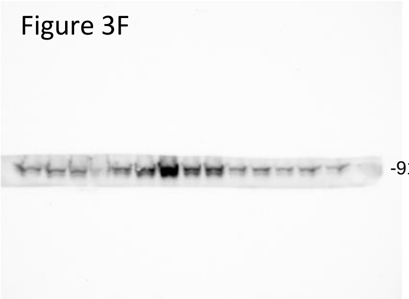

-91kD STAT1

a. B10.Q b. B10.Q.*Ncf1*$^{m1j/m1j}$ c. *Ncf1*$^{m1j/m1j}$.MN^{Tg}

a b c

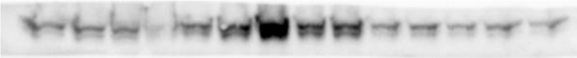

-91kD STAT1

28603 25037 24036 15417 24970 39612 69615 35669 38474 15933 17545 11457 16671 12031

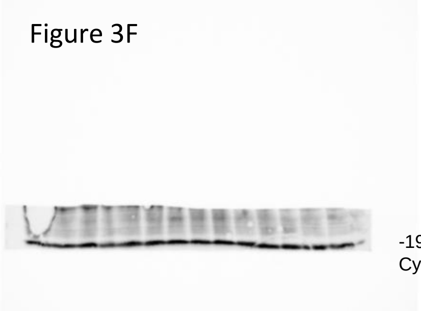

-19kD
Cyclophilin A

a. B10.Q b. B10.Q.*Ncf1*$^{m1j/m1j}$ c. *Ncf1*$^{m1j/m1j}$.MN^{Tg}

a b c

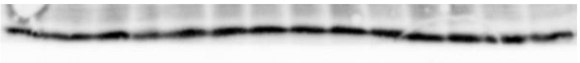

-19kD
Cyclophilin A

139258 131512 135871 105444 135774 164596 160623 152773 166614 122174 137848 115636 130653 112267

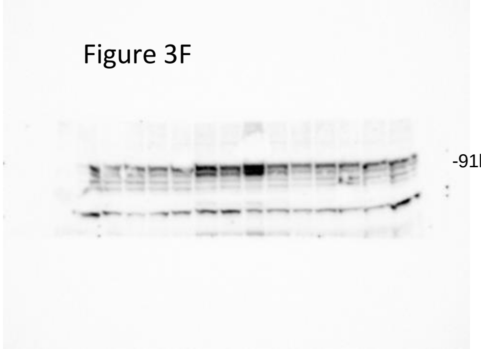

-91kD pSTAT1^{Tyr701}

a. B10.Q b. B10.Q.*Ncf1*$^{m1j/m1j}$ c. *Ncf1*$^{m1j/m1j}$.MN^{Tg}

a b c

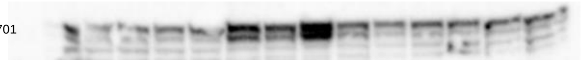

-91kD
pSTAT1^{Tyr701}

18930 12994 18073 22975 22218 60958 46017 80155 36663 27168 33630 32644 38849 45810

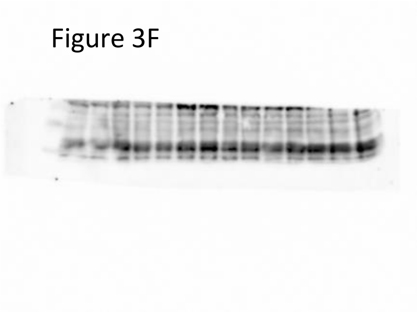

-19kD
Cyclophilin A

a. B10.Q b. B10.Q.*Ncf1*$^{m1j/m1j}$ c. *Ncf1*$^{m1j/m1j}$.MN^{Tg}

a b c

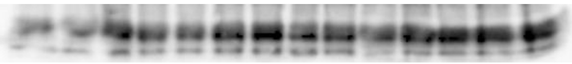

-19kD
Cyclophilin A

35888 31883 53014 44771 40533 57372 67149 49809 55873 52197 59774 57582 70605 74638

Figure 7E

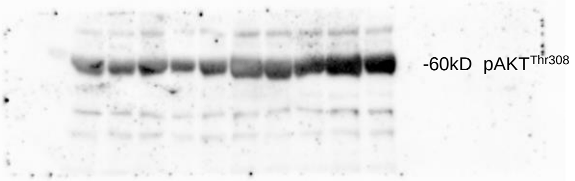

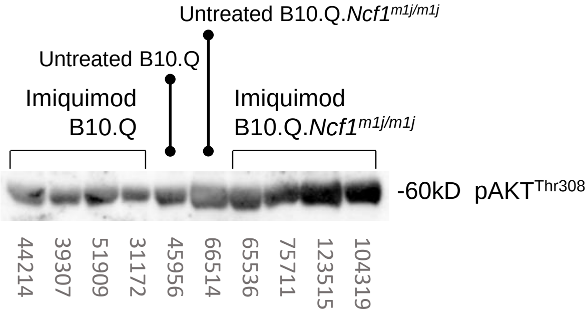

Figure 7E

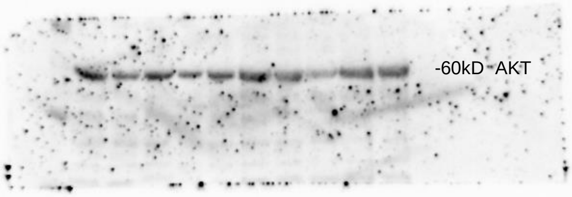

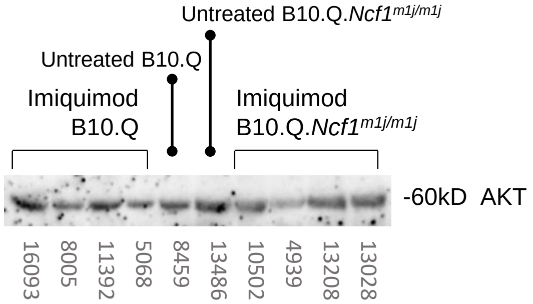

Figure 7E

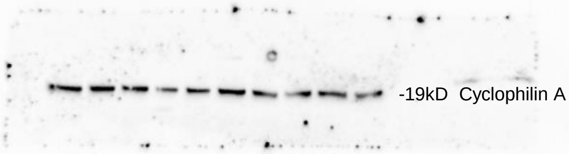

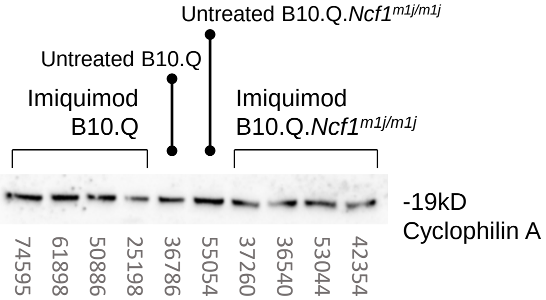

Figure 7E

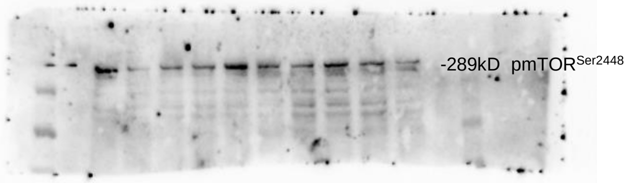

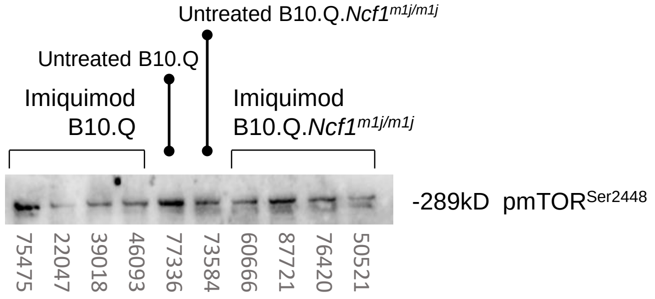

Figure 7E

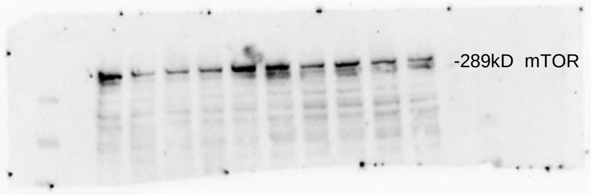

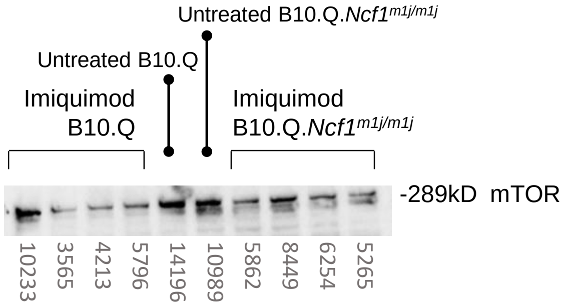

Figure 7E

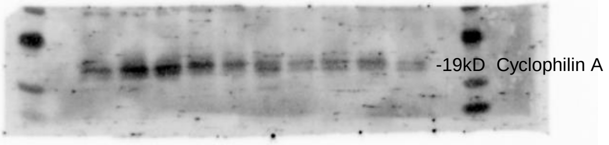

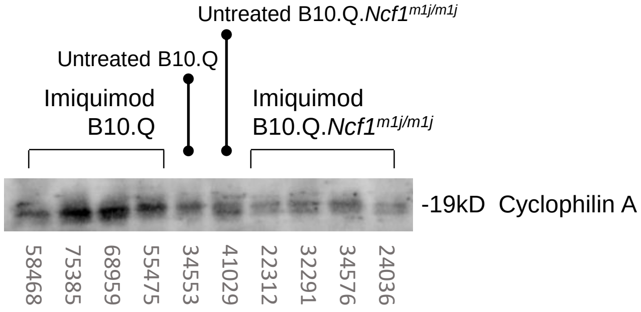

1: Untreated B10.Q
2: Untreated B10.Q.*Ncf1*$^{R90/90H}$
3: Imiquimod B10.Q
4: Imiquimod B10.Q.*Ncf1*$^{R90/90H}$
5: Imiquimod+20μM GSK2795039 B10.Q
6: Imiquimod+40μM GSK2795039 B10.Q
7: Imiquimod+10μM H_2O_2 B10.Q.*Ncf1*$^{R90/90H}$
8: Imiquimod+20μM H_2O_2 B10.Q.*Ncf1*$^{R90/90H}$

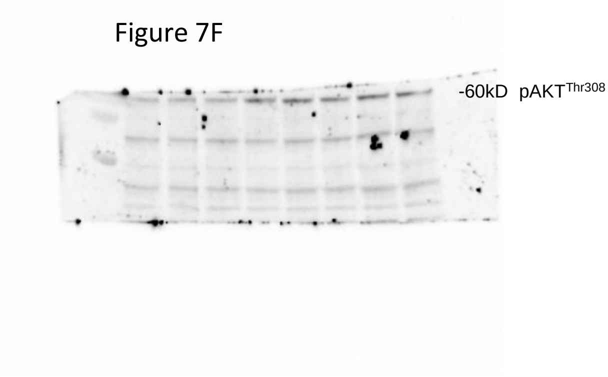

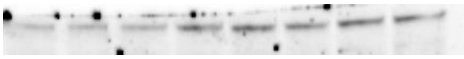

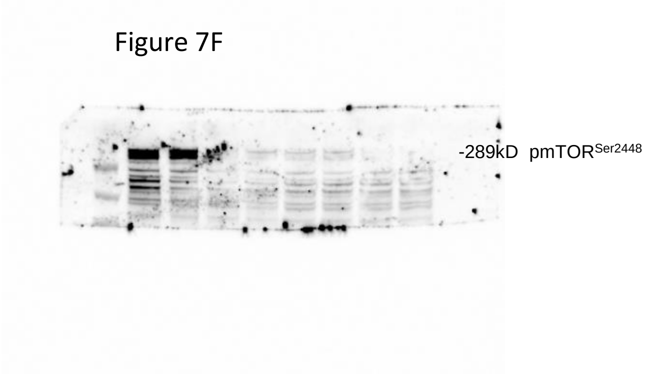

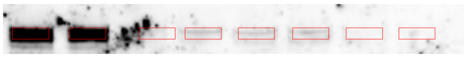

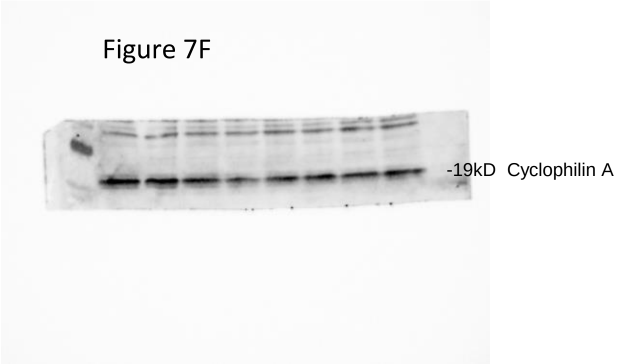

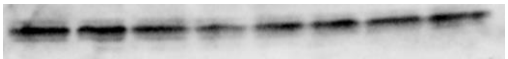

1: Untreated B10.Q
2: Untreated B10.Q.*Ncf1*$^{R90/90H}$
3: Imiquimod B10.Q
4: Imiquimod B10.Q
5: Imiquimod B10.Q.*Ncf1*$^{R90/90H}$
6: Imiquimod B10.Q.*Ncf1*$^{R90/90H}$
7: Imiquimod+40μM GSK2795039 B10.Q
8: Imiquimod+40μM GSK2795039 B10.Q
9: Imiquimod+20μM H_2O_2 B10.Q.*Ncf1*$^{R90/90H}$
10: Imiquimod+20μM GSK2795039 B10.Q
11: Imiquimod+20μM H_2O_2 B10.Q.*Ncf1*$^{R90/90H}$
12: Imiquimod+20μM GSK2795039 B10.Q

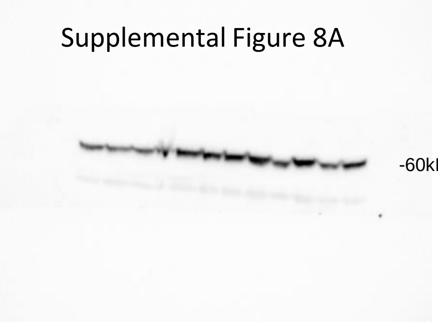

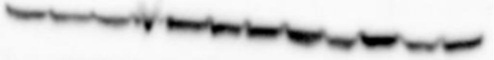

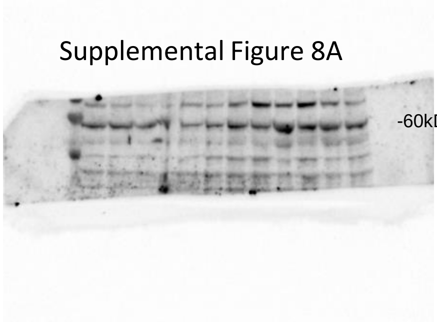

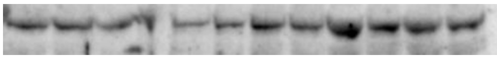

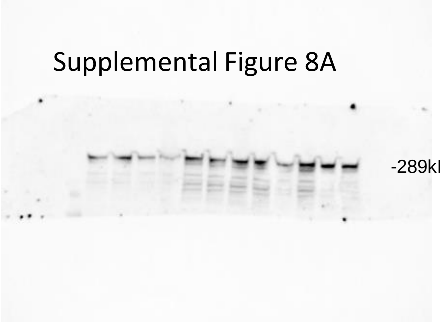

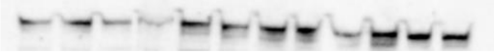

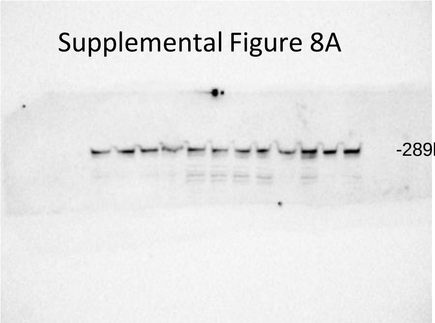

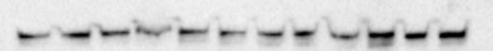

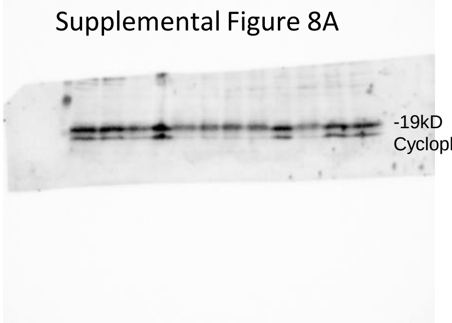

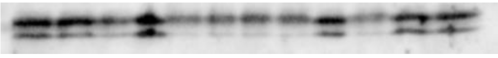

Supplement: Supplemental data [file jciinsight-8-164875-s060.pdf]
